# Reproductive traits associated with species turnover of amphibians in Amazonia and its Andean slopes

**DOI:** 10.1002/ece3.2862

**Published:** 2017-03-14

**Authors:** Octavio Jiménez‐Robles, Juan M. Guayasamin, Santiago R. Ron, Ignacio De la Riva

**Affiliations:** ^1^ Department of Biodiversity and Evolutionary BiologyMuseo Nacional de Ciencias NaturalesConsejo Superior de Investigaciones CientíficasMadridSpain; ^2^ Zoology DepartmentUniversidad de GranadaGranadaSpain; ^3^ BIÓSFERALaboratorio de Biología EvolutivaColegio de Ciencias Biológicas y AmbientalesUniversidad San Francisco de QuitoQuitoEcuador; ^4^ Centro de Investigación de la Biodiversidad y Cambio ClimáticoIngeniería en Biodiversidad y Recursos GenéticosFacultad de Ciencias de Medio AmbienteUniversidad Tecnológica IndoaméricaQuitoEcuador; ^5^ Museo de Zoología, Escuela de Ciencias BiológicasPontificia Universidad Católica del EcuadorQuitoEcuador

**Keywords:** assembly mechanisms, habitat filter, nestedness dissimilarity, species richness, trait–environment relationships, β‐diversity

## Abstract

Assembly of ecological communities is important for the conservation of ecosystems, predicting perturbation impacts, and understanding the origin and loss of biodiversity. We tested how amphibian communities are assembled by neutral and niche‐based mechanisms, such as habitat filtering. Species richness, β‐diversities, and reproductive traits of amphibians were evaluated at local scale in seven habitats at different elevation and disturbance levels in Wisui Biological Station, Morona‐Santiago, Ecuador, on the foothills of the Cordillera del Kutukú; and at regional scale using 109 localities across evergreen forests of Amazonia and its Andean slopes (0–3,900 m a.s.l.). At local scale, species composition showed strong differences among habitats, explained mainly by turnover. Reproductive modes occurred differently across habitats (e.g., prevalence of direct developers at high elevation, where breeding in ground level water disappears). At regional scale, elevation was the most important factor explaining the changes in species richness, reproductive trait occurrences, and biotic dissimilarities. Species number in all groups decreased with elevation except for those with lotic tadpoles and terrestrial reproduction stages. Seasonality, annual precipitation, and relative humidity partially explained the occurrence of some reproductive traits. Biotic dissimilarities were also mostly caused by turnover rather than nestedness and were particularly high in montane and foothill sites. Within lowlands, geographic distance explained more variability than elevation. Habitat filtering was supported by the different occurrence of reproductive traits according to elevation, water availability, and breeding microhabitats at both scales, as well as other assembly mechanisms based in biotic interactions at local scale. Human‐generated land use changes in Amazonia and its Andean slopes reduce local amphibian biodiversity by alteration of primary forests and loss of their microhabitats and the interaction network that maintains their unique amphibian assemblages with different reproductive strategies.

## 1 Introduction

Amazonian rainforests are considered the biota with the vastest diversity on Earth (Malhado et al., 2013). Endemism and species richness of several groups of organisms increase with the transition toward higher elevations and nutrient‐rich soils in Western Amazonia (Hoorn et al., 2010; Swenson et al., 2012). Different non‐exclusive hypotheses could explain this high diversity such as, for example, niche‐based processes that promote higher species richness due to higher habitat heterogeneity (Smith, Nieto Montes De Oca, Reeder, & Wiens, 2007), or certain conditions favoring higher ecosystem carrying capacities (Currie et al., 2004); and stochastic processes in which distribution ranges overlap more in the intermediate areas between natural boundaries (e.g., coasts, treeline) by pure randomness (mid‐domain effect hypothesis; Colwell, Rahbek, & Gotelli, 2004).

Assemblages are formed by processes of speciation, extinction, and dispersal, which are influenced by abiotic and biotic factors (Wiens, Pyron, & Moen, 2011). Differences in species composition can be described by two antithetic measures: turnover (replacement of some species by others) and nestedness (species loss or gain) (Baselga, 2010). Assembly is thought to follow some rules within several non‐exclusive mechanisms that could be niche‐based, historical (Baselga, Gómez‐Rodríguez, & Lobo, 2012), or neutral (*sensu* Hubbell, 2001). According to a niche‐based assembly rule called habitat templets, environment filters local species composition, according to their ecological niche, and promotes the origin and maintenance of particular natural history traits in communities (Southwood, 1977).

Environmental heterogeneity in Amazonian rainforests accommodates different habitats with particular associated groups of organisms, even in small areas (Tuomisto et al., 1995). The effect of habitat heterogeneity on Amazonian amphibian assemblages is still scarcely known (von May et al., 2010). Some studies suggest that amphibian communities are not homogeneous throughout Amazonia due to habitat diversity (e.g., Doan & Arizábal, 2002). Furthermore, diversity of amphibians may vary as a consequence of the availability of key habitat requirements (Almeida‐Gomes & Rocha, 2015; Bickford et al., 2010; von May et al., 2010). Therefore, local features may act as a habitat templet, favoring particular natural history traits in amphibian communities (Ernst et al., 2012). However, these research questions are generally addressed with regional species pools (Carstensen, Lessard, Holt, Krabbe Borregaard, & Rahbek, 2013) at a large scale that misses the local effect of habitat features on actual assemblages (von May et al., 2010).

The structural complexity of tropical rainforests offers a considerable variety of breeding microhabitats for coexistent amphibians (Haddad & Prado, 2005). Amphibians have a remarkable diversification of reproductive modes in the neotropics (Gómez‐Mestre, Pyron, & Wiens, 2012; Haddad & Prado, 2005). Reproductive modes diversity varies with factors such as humidity (Silva, Almeida‐Neto, do Prado, Haddad, & de Cerqueira Rossa‐Feres, 2012) and forest fragment size (Almeida‐Gomes & Rocha, 2015). Particular reproductive modes, such as direct development, foam nests, or lotic tadpoles, have been proposed as adaptations to certain climatic and biotic habitat features (Duellman, 1988; Duellman & Thomas, 1996; Gómez‐Mestre et al., 2012; Hödl, 1990).

Evergreen forests in the Amazon Basin reach elevations above 3,000 m in the eastern Andean montane forests (Gradstein, Homeier & Gansert, 2008). Although the forests of the Amazonian slopes are a conservation priority area, they have been reduced to less than one‐fourth of their original extension (Myers, Mittermeier, Mittermeier, da Fonseca, & Kent, 2000). The numerous taxonomic studies of amphibians of the Amazonian slopes (e.g., Duellman & Lehr, 2009; Guayasamin & Funk, 2009; Lynch & Duellman, 1980; Padial et al., 2012; Páez‐Moscoso & Guayasamin, 2012) contrast with the few intensive studies of their assemblages. Herein, we analyze the variation of amphibian communities in terms of species richness, composition, and occurrence of reproductive traits at two different scales: a local study in the northeastern foothills of Cordillera del Kutukú, Ecuador, and a compilation of some of the most complete amphibian inventories of the Amazonian lowlands and the Western Amazonian slopes. At local scale, we expected variation according to the habitat type and its availability of breeding microhabitats, along disturbance and elevation gradients. At a broader scale, we were intrigued about the variation of species richness, biotic dissimilarities, and reproductive traits occurrence regarding environmental features, as well as the existence of foothill and montane endemisms. In addition, we discuss the relevance of our results for the conservation of Amazonian ecosystems and, more concisely, of the Amazonian slopes of the Andes.

## 2 Methods

### 2.1 Area of study

At local scale, our study was carried out in Wisui Biological Station, in the Cordillera del Kutukú, municipality of Makuma, Morona‐Santiago, Ecuador (02°06′39″S, 77°44′19″W). This mountainous area is predominantly covered by pristine foothill evergreen forest. All sampled habitats occurred at elevations of 600–800 m, excepting primary forest that extended from 600 to 1,400 m (Fig. S1).

### 2.2 Data collection

Six visits to Wisui were made from December 2008 to February 2010, accounting for a total of 50 sampling days: 18 December 2008–1 January 2009, 20 February–2 March, 22–28 March, 24–29 May, 7–11 December 2009, and 18–23 February 2010.

Surveys were stratified in seven different habitats: anthropogenic habitats; artificial pools; riverine habitats; secondary forest; and three kinds of primary forest, subcategorized by elevation ranges as low (600–800 m), intermediate (800–1,100 m), and high (1,100–1,400 m). A more detailed description of habitats can be found in Appendix S1. Occurrence of reproductive sites in every habitat was recorded. We found nine types of reproductive sites: rivers (>3 m width), wide streams (2–3 m width), small streams (<2 m width), rain puddles, ponds, bromeliads, leaf litter, extensive moss cover, and leaf‐cutter ant nests (Table S1).

The main sampling method was time‐constrained visual encounter surveys (VES; Crump & Scott, 1994) along paths and around pools, both in daytime and nighttime. While visually looking for any evidence of amphibians, and directing efforts to find and identify unrecognized calling anurans, we always quantified the sampling time spent in each habitat. Periodically, we evaluated the completeness for every habitat inventory calculating its sample coverage (Chao & Jost, 2012) from the registered number of species and their abundances, using the R package iNEXT (Hsieh, Ma, & Chao, 2016). To avoid strong differences among the completeness of the assemblages, we invested more effort (measured in person‐hours) in the habitats with the lowest sample coverages. VES data were complemented with incidental observations. Voucher specimens (Table S2) were deposited in the collections of Pontificia Universidad Católica del Ecuador (QCAZ) and Universidad Tecnológica Indoamérica (MZUTI).

### 2.3 Local data analysis

The sample coverages obtained for the final sample sizes were not evenly distributed. Therefore, for comparing species richness, we extrapolated them and their 95% confidence intervals to an equal sample coverage following the extrapolation method (detailed in Chao & Jost, 2012) performed in iNEXT (Hsieh et al., 2016) with 100 randomizations. That particular sample coverage is called base sample coverage, and it is chosen as the largest of either the lowest coverage for doubled sample sizes or the maximum coverage for original sample sizes (Chao & Jost, 2012).

Differences in species composition (β‐diversity) among assemblages were assessed through pairwise Sørensen dissimilarities and its turnover and nestedness components using betapart (Baselga & Orme, 2012), non‐Metric Multidimensional Scaling (nMDS), and counting the number of unique species (those present in only one habitat). In order to simplify analyses, we integrated the species detected opportunistically in the presence dataset obtained through VES for each habitat.

We grouped the species found in Wisui according to their reproductive mode (Haddad & Prado, 2005), and we obtained data on their clutch sizes consulting the literature of Amazonian amphibians ecology (see Appendix S2). In the case of species with no previous reported clutch size, we estimated it following Duellman (2005) and Duellman and Lehr (2009) SVL correlations. We assessed the occurrence of each reproductive mode within and between habitats through Shannon diversities, which integrates both species number and frequencies, without favoring either common or rare species (Jost, 2007). Again, we observed their mean and 95% confidence intervals in an equal sample coverage (following Chao et al., 2014) randomizing 100 times with iNEXT (Hsieh et al., 2016).

### 2.4 Biogeographic comparisons

We compiled amphibian species lists from other highly complete inventories (books, articles, checklists, and museum collection databases, detailed in Table S2). Although the elevational limits of different forests may vary with latitude along the Amazonian slopes, for the sake of simplicity, we classified the localities as lowland (0–400 m), foothill (400–1,200 m), and montane (1,200 m–treeline).

We accounted for unique species and multisite β‐dissimilarities for each elevational group with equal sample size (following Baselga, 2010). With the pairwise biotic dissimilarities we also applied Generalized Dissimilarity Modeling (GDM), a statistical technique extended from matrix regressions (Ferrier, Manion, Elith, & Richardson, 2007) to measure the contribution of geographic distance and a set of ecological variables. We included elevation, annual precipitation, precipitation seasonality (extracted from WorldClim; Hijmans, Cameron, Parra, Jones, & Jarvis, 2005), and mean annual relative humidity (averaged from New, Lister, Hulme, & Makin, 2002 layers). Temperature variables were not included because of their high colinearity with elevation. We performed the GDMs in the gdm R package (Manion, Lisk, Ferrier, Nieto‐Lugilde, & Fitzpatrick, 2016) among all the localities and separately within lowland, foothill, and montane sites.

We quantified in each inventory how many species have the following non‐exclusive reproductive traits: oviposition at ground level water, phytotelm tadpoles, lotic tadpoles, lentic tadpoles, direct development, terrestrial eggs (e.g., Centrolenidae, Dendrobatidae, all direct developers), and terrestrial metamorphosis (e.g., *Adenomera*,* Synapturanus*, all direct developers), consulting relevant sources (iucnredlist.org and references in Appendix S2). In some cases, when information on reproductive traits was absent, we assumed the species had the same reproduction mode as other phylogenetically closely related species, if the trait is consistent in that clade (e.g., direct development in Craugastoridae or foam nests in Leptodactylidae). Some species had to be excluded because their specific reproduction is unknown and it varies within their species group (e.g., *Dendropsophus* and *Hypsiboas* may lay eggs on water or vegetation, and their tadpoles may develop on lentic or lotic waters). Because total amphibian richness and the number of species of every type of reproduction are positive whole numbers, we used negative binomial Generalized Linear Models (GLMs). We selected the best GLM by stepwise variable reduction based in Akaike Information Criteria and correcting overdispersion by an adjusted **θ** parameter (glm.nb and stepAIC functions in MASS package; Venables & Ripley, 2002). Predictor selection started with the same set of variables used in the GDMs and a quadratic term for elevation given its potential non‐linear relation with Amazonian amphibian diversity (Hutter, Guayasamin, & Wiens, 2013; Smith et al., 2007).

## 3 Results

### 3.1 Local inventory completeness and richness

In Wisui, we recorded 56 species of amphibians (two caecilians, two salamanders, 52 anurans; Table S2). After 274 person‐hours of VES, 449 individuals of 51 species were found. The other five species were registered opportunistically.

According to estimated and extrapolated species richness (Table 1), there are species still to be found in most habitats of Wisui. Incidental captures confirmed 17 species in different habitats (Table 1). Sample coverage of VES was 0.98 (pooling all the habitats together), and ranged 0.79–0.98 individually in habitats (Table 1). As the maximum coverage (0.98 for artificial pools) was higher than the lowest coverages for doubled sample sizes (0.83 for riverine habitats and 0.87 for low primary forest), richness estimations were extrapolated to a standard base sample coverage of 0.98 (Chao & Jost, 2012; Chao et al., 2014). Confidence intervals were wider in the assemblages with lower completeness, impairing finding significant differences in richness excepting some pairwise comparisons (e.g., among artificial pools, secondary, and high primary forest; among artificial pools, low, and high primary forest; and anthropogenic habitats against secondary, and low primary forest; Table 1). Excluding riverine habitats due to a possible mathematical artifact (see comments in Table 1), the low primary forest assemblage was first in mean extrapolated species richness at common sample coverage (Chao & Jost, 2012).

**Table 1:** Sampling effort and results in the amphibian assemblages of Wisui Biological Station. See Section 3 for explanation

Habitats	Visual encounter surveys (VES)						Add. S_obs_
	Sampling effort (person‐hours)	Ind	S_o_	S_e_ ± *SE*	C	S (C_0.98_) ± CI	
AH	34	21	7	9.9 ± 4.2	0.86	9.5 ± 4.9	3
AP	28	115	13	14 ± 2.2	0.98	13 ± 1.7	−
RH	32	47	14	62.9 ± 58.3^a^	0.79^a^	58.9 ± 50.4^a^	1
SF	54	74	19	29 ± 8.9	0.88	27.5 ± 11.3	4
LPF	54	48	12	43.3 ± 38.8	0.83	40 ± 23.9	4
IPF	38	63	11	16.9 ± 7	0.94	15.3 ± 6.7	1
HPF	34	84	6	8 ± 3.7	0.98	6.5 ± 2.4	−
Total (γ‐diversity)	274	451	51	73.5 ± 16	0.98	61.9 ± 10.4	5

Ind, observed individuals; S_o_, observed species through VES; S_e_, estimated richness through Chao 1 estimator (Chao 1984); C, sample coverage; S(C_0.98_), estimated richness at the base sample coverage; Add. S_o_, additional species registered through incidental captures.

a We re‐analyzed the riverine habitats assemblage data subtracting the four single records of taxa we think that appeared incidentally as a result of a pitfall effect of the Tayuntza river gorge on the vicinity low primary forest (*Caecilia* sp., *Rhaebo ecuadorensis*,* Ranitomeya variabilis*,* Pristimantis rubicundus*). The recalculated estimated and extrapolated richness had more sensible values (S_e_ ± *SE* = 27.6 ± 23.1; C = 0.84; S (C_0.98_) ± CI = 25.4 ± 21.2), and left low primary forest with the lowest sample coverage.

### 3.2 Local β‐diversity, species composition and reproductive traits

**Figure 1:**
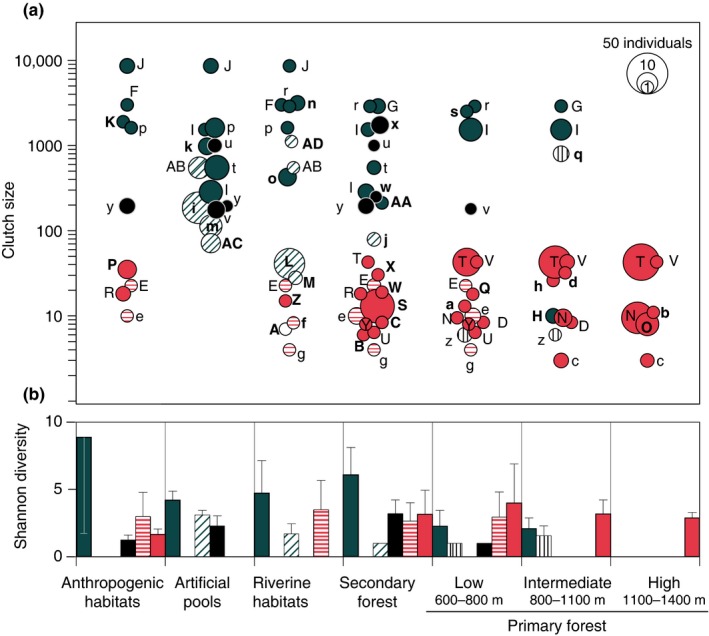
(a) Assemblages of amphibians and their reproductive traits in Wisui Biological Station, Morona‐Santiago, Ecuador. Abundance of each species is represented by circle size on a logarithmic scale. Clutch size is represented by the position along the *y*‐axis. Letters representing each species are summarized in Table S1. Bold letters correspond to habitat unique species. (b) Shannon diversities of reproductive mode groups in the same assemblages. Mean values and 95% confidence intervals are calculated for equal sample coverage of 0.94 (following Chao & Jost, 2012 protocol). Colors represent reproductive mode groups in both graphs: solid dark blue, eggs laid in water bodies at ground level; diagonally‐hatched blue, eggs laid on vegetation overhanging water; vertically‐hatched black, eggs laid on *phytotelmata*; solid black, eggs in foam nests; horizontally‐hatched red, eggs laid on ground but larvae transported by adults to water; solid light red, direct development; white, unknown reproduction

Every assemblage in Wisui has different species composition and unique species (Figure 1). Thirty‐three of the 56 species were found in a single habitat, and 19 of these were found only once. The most common species of most habitats (excepting primary forest) were unique of them (e.g., *Oreobates quixensis* in anthropogenic habitats, *Dendropsophus bifurcus* in artificial pools, *Rulyrana flavopunctata* in riverine habitats, and *Pristimantis altamnis* in secondary forest). The *Pristimantis conspicillatus* complex was dominant in all the elevations of primary forest and almost absent in secondary forest. Although different elevations of primary forest shared some taxa, their abundances changed along elevation (e.g., *Rhinella margaritifera*,* Pristimantis trachyblepharis*,* P. diadematus*, and *Hypodactylus nigrovittatus*; Figure 1).

**Figure 2:**
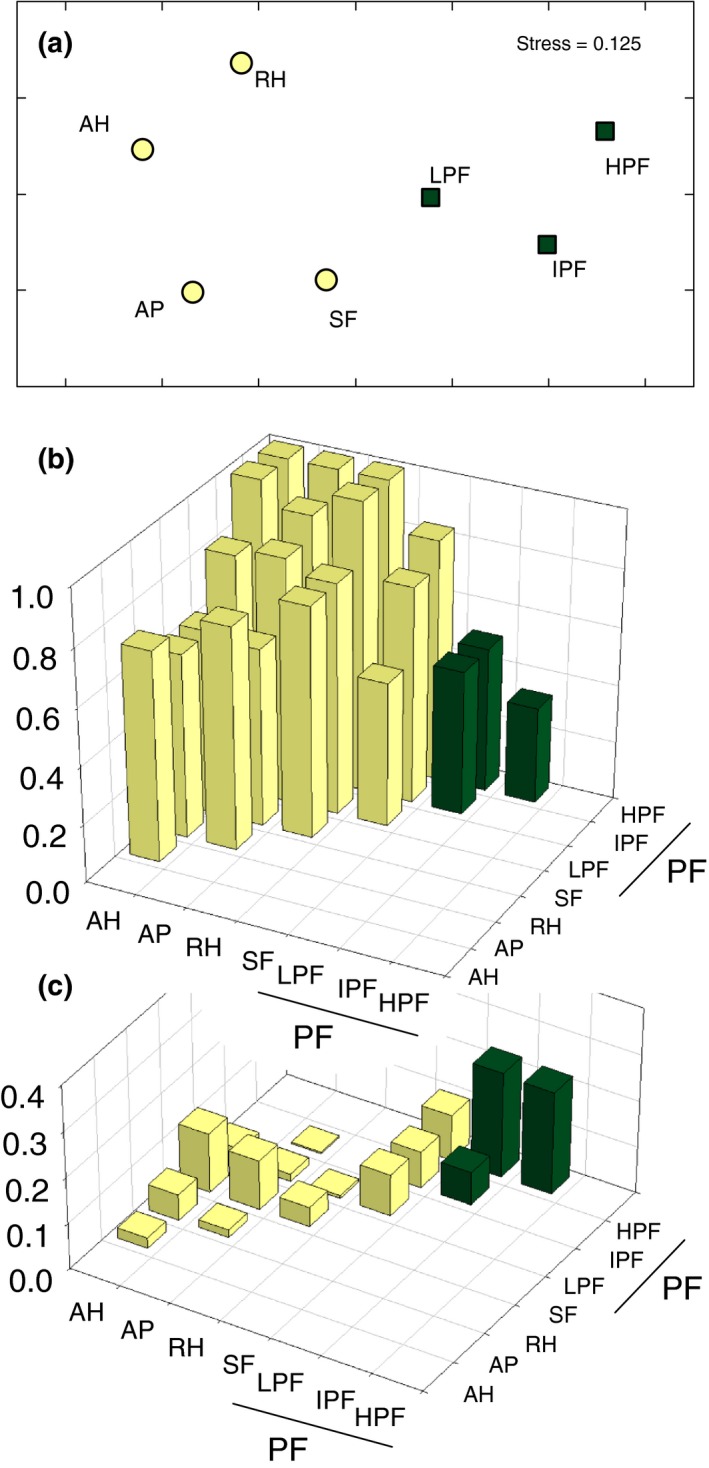
β‐diversities among the assemblages of amphibians found in Wisui Biological Station (VES and incidental observations combined): anthropogenic habitats (AH), artificial pools (AP), riverine habitats (RH), secondary forest (SF), low primary forest (LPF), intermediate primary forest (IPF), and high primary forest (HPF). (a) Non‐Metric Multidimensional Scaling ordination based in pairwise Sørensen dissimilarities. Dark green squares represent primary forest, while the rest of habitats are the yellow circles. Additive decomposition of β‐diversity: (b) turnover (Simpson dissimilarities) and (c) nestedness dissimilarities. Dark green bars represent the results within primary forest habitats, and yellow bars the rest of pairwise comparisons

According to dissimilarities (Figure 2), all the assemblages of amphibians were distinct, although the three primary forests were more similar than the rest. The secondary forest assemblage remained in a neutral position centered among primary forests and other habitats. As expected from the amount of unique species, turnover explained most of the incidence‐based differences among the assemblages (60%–100%; Figure 2b). Nestedness dissimilarity was also considerable at different elevations of primary forest, reaching up to 40% of incidence‐based β‐diversity between intermediate primary forest and high primary forest (Figure 2c). In these cases, turnover contribution was smaller than among the rest of assemblages.

In Wisui, we recorded 13 reproductive modes *sensu* Haddad and Prado (2005), but we clustered them in seven groups (Table S2). Clutch sizes differed between these groups and their diversities across habitats were unevenly distributed (Figure 1). The ancestral reproductive mode (eggs laid in water at ground level) was present in all habitats except high primary forest, being predominant in anthropogenic and aquatic habitats (artificial pools, riverine habitats) and exhibiting the biggest clutch sizes. Eggs hanging from vegetation above the water were the most frequent mode in artificial pools, occurred in riverine habitats, and were present in secondary forest. Egg deposition in *phytotelmata* was found in low primary forest and with higher frequency at intermediate primary forest. Species with foam nests had the second largest clutches and occurred at low densities in anthropogenic habitats, artificial pools, and secondary forest. Eggs laid on ground and larvae transported by adults to water (e.g., Aromobatidae and Dendrobatidae) had some of the lowest clutch sizes and occurred in anthropogenic habitats, riverine habitats, secondary forest, and low primary forest. Direct development, always with small clutches, was present in all habitats except artificial pools, and it became dominant with increasing elevation being the only strategy found in high primary forest.

### 3.3 Biogeographic analysis

We compiled species lists of 109 inventories: 57 lowland, 22 foothill, and 30 montane sites (Fig. S1 and Table S3) that included 5,077 presences of 897 taxa. Up to 467 amphibians were unique from single localities in our analysis, and 302 belonged to unidentified species. At least 198 described species of amphibians were unique for a single site (51, 21, and 126 for lowland, foothill, and montane localities, respectively). Oviposition and metamorphosis microhabitats were unknown for 288 and 167 species respectively, so we excluded them from the analyses of reproductive traits occurrence.

**Figure 3:**
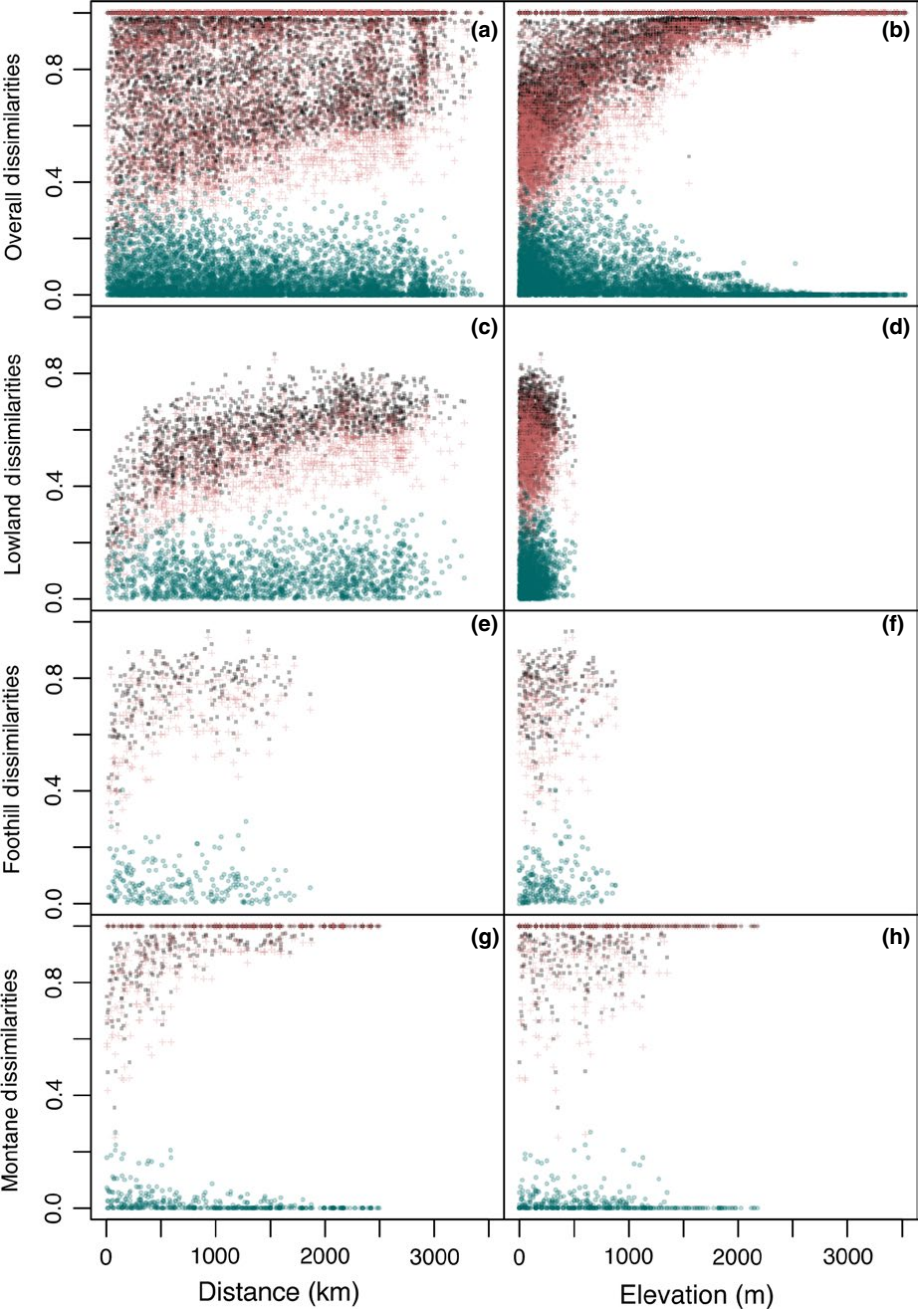
Pairwise β‐dissimilarities against geographic distance (left column) and elevation difference (right column) for (a, b) all the sites (from sea level to treeline), (c,d) lowland (0–400 m), (e,f) foothill (400–1,200 m), and (g,h) montane sites (1,200 m–treeline) included in our analyses of Amazonian amphibian assemblages. Sørensen dissimilarities are represented by black spots, while their additive components are represented in orange (turnover or Simpson dissimilarity) and blue (nestedness dissimilarity)

**Figure 4:**
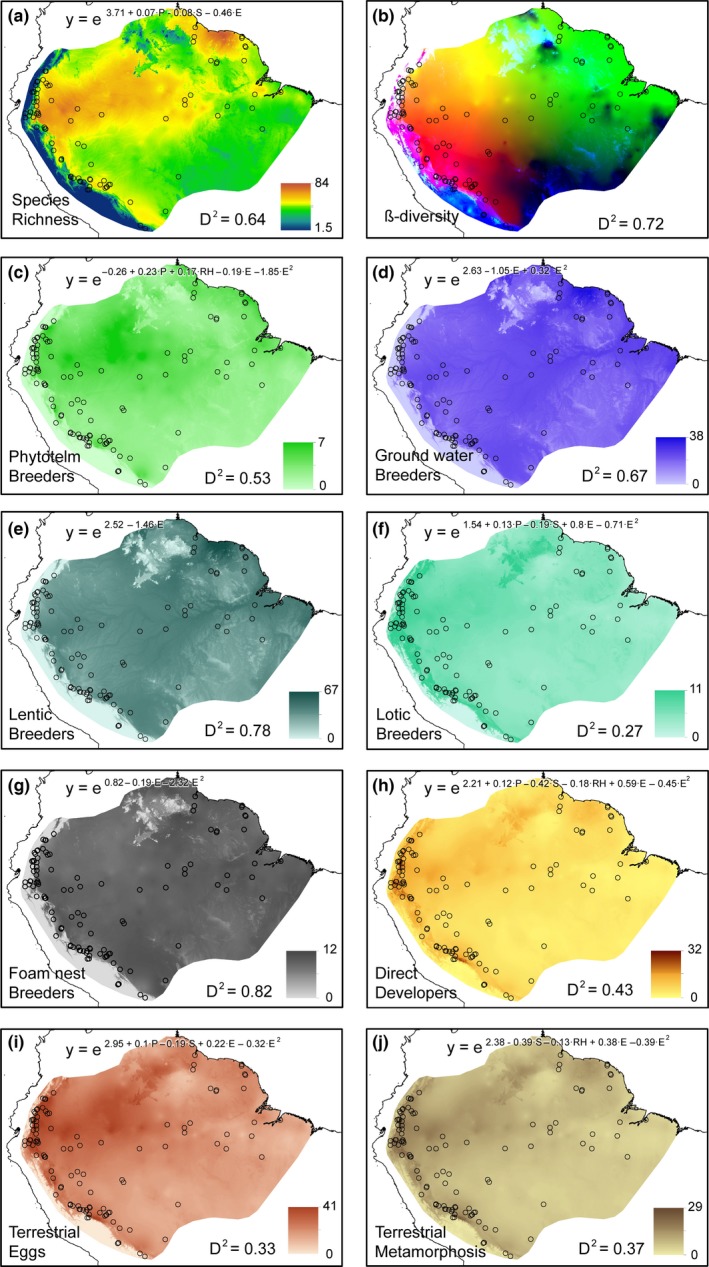
Maps representing the predictions of the models for: (a) Species richness, (b) β‐diversity (Sørensen Dissimilarity), (c) phytotelm breeders, (d) species with tadpoles in ground level water, (e) species with lentic tadpoles, (f) species with lotic tadpoles, (g) species with eggs in foam nests, (h) direct developers, (i) terrestrial eggs, and (j) terrestrial metamorphosis. In the map of β‐diversity, the more similar colors characterize similar assemblages. Explained deviance and model equations are detailed, except the latter in the case of the Sørensen GDM

The main component of β‐diversities was turnover, as Simpson dissimilarities were really close to Sørensen dissimilarities (Table 2 and Figure 3). The GDM model including all the localities explained 83.8%, 82%, and 2.3% of the total deviances for Sørensen, Simpson, and nestedness dissimilarities, respectively (Table 2 and Figure 4b). Approximately half of the explained deviance of Sørensen and Simpson dissimilarities among all sites was captured by elevation, a 3% by geographic distance, while no more than 1% could be attributed to the rest of predictors. However, elevation did not explain more than 2% of variation of any biotic dissimilarity among lowlands and up to 10% in the best case among foothill or montane areas. Contrarily, geographic distance explained up to 37%, 14%, and 11% of lowland, foothill, and montane β‐diversities, respectively.

**Table 2:** Multisite dissimilarities and the explained deviances of the GDMs models (D^2^) and the each one of the predictors: annual precipitation (P), precipitation seasonality (S), and elevation (E), relative humidity (RH), and geographic distances (G)

Variable	Multisite	D^2^	Fraction of null deviance purely explained by				
	β‐diversity		P	S	RH	E	G
**Sørensen dissimilarity**							
All	0.93	0.84	0.01	0.006	0.001	0.41	0.03
Lowlands	0.89	0.64	0.01	0.027	0.004	0.02	0.37
Foothills	0.92	0.45	0.01	0.006	0.000	0.09	0.14
Montane	0.97	0.61	0.00	0.004	0.001	0.10	0.11
**Simpson dissimilarity (turnover)**							
All	0.89	0.82	0.01	0.007	0.004	0.393	0.03
Lowlands	0.85	0.51	0.005	0.029	0.010	0.003	0.30
Foothills	0.89	0.41	0.03	0.019	0	0.045	0.13
Montane	0.95	0.58	0	0.003	0.002	0.090	0.11
**Nestedness dissimilarity**							
All	0.04	0.02	0.013	0	0.006	0	0
Lowlands	0.04	0.03	0.005	0	0	0.01	0
Foothills	0.03	0.04	0.012	0	0.014	0.01	0
Montane	0.02	0	0	0	0	0	0

Richness was better explained (64% of total deviance) by a GLM including elevation (monotonic), annual precipitation, and precipitation seasonality (Table 3 and Figure 4). The selected GLMs for reproductive traits explained 27%–82% of total deviance (Table 3 and Figure 4). In all of them, elevation had a significant effect and explained 2%–78% of total deviance, with a negative effect, except for direct developers, lotic breeders, terrestrial eggs, and terrestrial metamorphosis. Most of the GLMs included a negative quadratic effect with elevation (which means a decrease in mid elevations), except in the ancestral mode (eggs deposited in ground‐level water), which had a positive quadratic effect (mid‐elevation increase), and both species richness and lentic breeders with no quadratic effect. Precipitation seasonality explained 4%–17% of data variability in lotic breeders, direct developers, terrestrial eggs, and terrestrial metamorphosis, with a negative effect except for the latter group. Annual precipitation and relative humidity, when selected, explained 1%–2% of total variability, always with a positive effect in the former and with different coefficient sign in the latter.

**Table 3:** Data variability explained by negative binomial GLMs (D^2^) and their predictors for the species richness and occurrence of amphibians with particular reproductive traits in 57 lowland, 22 foothill, and 30 montane Amazonian localities (detailed in Table S3). The considered predictors were selected by a stepwise procedure starting from: annual precipitation (P), precipitation seasonality (S), relative humidity (RH), and elevation (E). Significance estimates for all the models were corrected for heteroscedasticity by a Wald test with the “sandwich” correction (R packages lmtest, Zeileis & Hothorn, 2002; and sandwich, Zeileis, 2004), giving always a *p* value lower than .000001 and an overdispersion estimate Φ of 1–1.3. The standard coefficients for each variable in a model are detailed in Figure 4

Response variables	D^2^	Null deviance purely explained by			
		P	S	RH	E
**α‐diversity**					
Amphibian species richness^a^	0.64	0.01	0.01	–	0.40
**Reproductive traits occurrence**					
Phytotelm breeders	0.53	0.02	–	0.01	0.28
Eggs laid on ground water	0.67	–	–	–	0.67
Lotic breeders	0.27	0.01	0.04	–	0.09
Lentic breeders	0.78	–	–	–	0.78
Foam nests	0.82	–	–	–	0.82
Direct developers	0.43	0.01	0.12	0.02	0.04
Terrestrial eggs	0.33	0.02	0.07	–	0.03
Terrestrial metamorphosis	0.38	–	0.17	0.02	0.02

a The global species richness model excluded the nine localities in which surveyed extension was higher than in the others, resulting in higher levels of species richness per locality (see Table S3 for more details).

## 4 Discussion

Environment features have different effects depending on the reproductive traits of amphibians, suggesting that these strategies serve as adaptations to different opportunities along the evolutionary history of Amazonian and Andean amphibians.

The high species replacement among amphibian communities in the Amazon and its Andean slopes is driven by environmental filtering that can be observed at different spatial scales. On the one hand, coarse scale analyses are useful to explore correlations of regional species pools with climatic variables that are interpolated for big areas from a limited number of locations for long periods of time (Hijmans et al., 2005; New et al., 2002). On the other hand, fine scale analyses allow for detection of particular features that are key in the conformation of amphibian assemblages: the presence of structural elements, availability of breeding microhabitats, species interactions, etc. The combination of different spatial scales provides more insights to understand different assembly processes.

### 4.1 Local assembly processes

Although we reached 79%–98% sample coverages, and actual dissimilarities among Wisui habitats could be smaller than among our samples, just regarding the dominant taxa, species composition changes remarkably. Such differences in such a small area should rise concerns about assembly processes missed in broad‐scale diversity studies. Other studies on tropical amphibian assemblages at small spatial scales also showed strong differences in species composition (Das, Jankowski, Makmor, & Haas, 2007; Heyer, 1967; von May et al., 2010). Therefore, the high diversity of amphibians in the Amazon and other tropical areas may result from the contribution of different habitat types at every site.

In addition to the idiosyncrasy in species composition, the differences found also in occurrence of reproductive traits point out that communities are conformed only by species whose traits pass the filter imposed by the environment. Trait distribution patterns reflect the adaptation to temporal and spatial variability of the environment (Southwood, 1977). Species with the ancestral reproductive mode tend to lay big clutches, while other amphibians evolved toward modes with smaller clutch sizes but higher probability of progeny survival (Crump, 1974; Hartmann, Hartmann, & Haddad, 2010). In Wisui, we found that habitats with higher species diversity (primary and secondary forests) also have more evolved reproductive strategies, while disturbed habitats have more prevalence of high fecundity species. Similar patterns have been observed in other Amazonian localities (Crump, 1974; Duellman, 1978; Hödl, 1990) and the Brazilian Atlantic forest (Almeida‐Gomes & Rocha, 2015; Baselga & Orme, 2012; Haddad & Prado, 2005; Hartmann et al.*,* 2010; Silva et al., 2012). Duellman (1978) interpreted this variation in amphibian reproductive strategies as an adaptation to the stability and predictability of environments. Therefore, disturbance benefits species with higher fecundity (i.e., more ancestral reproductive modes), while stable and predictable habitats (such as primary forests) host more evolved reproductive modes.

Nonetheless, besides stability, habitat heterogeneity might be the main cause of high diversity of amphibians in tropical forests. In Wisui's assemblages, low elevation forested areas show the highest variety of breeding microhabitats, which could explain their higher number of reproductive modes and species richness. Primary *terra firme* forest is the habitat of Amazonia that hosts more reproductive modes, even compared with flooded primary habitats (Hödl, 1990). In other tropical regions, amphibian diversity of aquatic habitats exceeds that of forests (Ernst et al., 2012; Zimmerman & Simberloff, 1996). As observed in Wisui and other localities, Western Amazonia rainforests have high prevalence of specialized reproductive modes that make these amphibian assemblages more diverse than those of aquatic habitats. The structure of these neotropical rainforests, with such a variety of breeding microhabitats, is critical for hosting high diversities of amphibian species.

A considerable portion of the heterogeneity of amphibian breeding microhabitats in tropical rainforests can be attributed to niche constructors, organisms that alter the environment with evolutionary or ecological consequences for themselves or other species (Laland, Matthews, & Feldman, 2016). For example, the abundant bromeliads in Wisui's intermediate primary forest provide a unique breeding microhabitat for amphibians dependent on *phytotelmata* such as *Osteocephalus* aff. *fuscifacies* or *Chiasmocleis antenori*. Another conspicuous example is *Lithodytes lineatus,* which breeds inside leaf‐cutter ant nests (Schlüter, Löttker, & Mebert, 2009) and was more abundant in anthropogenic habitats and secondary forest in Wisui. These ants are more abundant in disturbed areas with secondary growth, where tender leaves are more common (Vasconcelos & Cherrett, 1995). Therefore, species interactions can be important in the assembly of amphibian communities, especially those with niche constructors that create breeding microhabitats, such as burrowing arthropods, or water‐containing plants.

The way in which organisms are filtered in different habitats does not only depend on structural features. Amphibians with direct development occur in all the terrestrial habitats of Wisui, even where other reproductive modes are impaired by the lack of standing ground water (e.g., intermediate and high primary forests). Prevalence of direct developers at intermediate elevations has been observed in other tropical areas (Das et al., 2007; Müller et al., 2013) and seems to be related with a higher humidity favored by topographic exposure to wet air masses. Therefore, local variation in non‐structural abiotic conditions with physiological importance, such as microclimates, might also contribute to the differential assembly of communities through habitat filtering.

In Wisui, turnover among different elevations of primary forest is lower than among other assemblages, although they also host unique species. At the same time, nestedness dissimilarity (in the form of species loss along elevation) is more important among elevational levels of primary forest than among the rest of habitats. This nested loss of species follows the usual amphibian species richness decrease with elevation (Das et al., 2007; Duellman, 1988). Although turnover is the main descriptor of β‐diversity of Amazonian amphibians at all scales, nestedness dissimilarity along elevation is more important at local scale than what we found among the sparse localities in our broad‐scale analysis.

### 4.2 Environmental filtering at regional scale

Elevation was one of the most important factors explaining variation in amphibian richness, reproductive traits occurrence, and species replacement. Beside the monotonic decrease in species richness with elevation, turnover (and not nestedness dissimilarity) was the main descriptor of β‐diversity among amphibian communities. As we commented above, nestedness dissimilarity may result more relevant at finer spatial scales. Elevation also had a negative effect on the occurrences of most of the reproductive traits, excepting lotic breeders and species with terrestrial reproduction stages, whose numbers increased non‐linearly with elevation. Therefore, elevation or other colinear ecological variables, such as temperature and habitat structure, are some of the most relevant elements structuring amphibian communities according to their influence on different reproductive traits.

Rainfall and seasonality also had a significant effect on regional species richness, with much less deviation explained than elevation. Other studies (e.g., Bass et al., 2010) also showed higher diversity of amphibians in the most aseasonal areas of the Amazonian rainforest, which are often some of the rainiest. Also, in lotic breeders and species with terrestrial reproduction stages, seasonality and precipitation or humidity explained larger parts of the deviances. As expected from amphibian physiology, several variables related to water availability (precipitation, humidity, and seasonality) are key factors structuring the diversity patterns of Amazonian amphibians at broad scale.

Species replacement across the Amazonian dataset increases along elevation. In fact, most of the amphibians found in the foothills or montane forests have limited distribution ranges, while lowland species ranges are wider in area and latitude. This is probably because similar ecological conditions are more contiguous in the lowlands, while in the slopes they are spatially divided by the complex topography, favoring allopatric speciation. In fact, the reproductive traits favored in foothills and montane habitats, such as direct development and lotic tadpoles, are principally represented by speciose taxa such as Terrarana, Centrolenidae, *Hyloxalus*,* Hyloscirtus*,* Colomascyrtus*, and *Atelopus*. Then, habitat filtering, summed to spatial heterogeneity, promotes an increase in β‐diversity and therefore, the overall species richness (γ‐diversity). Thus, the middle‐elevation peak exhibited by species richness (in the highest elevations of our lowland localities, e.g., Tiputini, Yasuní, Santa Cecilia) would not be explained by stochastic processes such as the mid‐domain effect but by environmental filtering.

Data shortfalls are a common issue in biogeographic studies of Amazonian diversity (Malhado et al., 2013), being the lack of systematic and intensive amphibian inventories even bigger in the Amazonian slopes. Consequently, with our limited dataset, the conclusions about such a vast area should be taken with caution. However, these limitations are compensated by the high sampling effort of the selected inventories and the inclusion of undescribed taxa (often excluded from macroecological studies), with an increase in robustness, as other less intensively sampled localities would be more affected by false absences. Also, the environmental conditions found in the selected localities are representative of the most frequent values observed at different elevations (Figs S2 and S3). Thus, the observed trends of habitat filtering are robust enough to describe part of the variation of diversity of amphibians and their natural history traits in Amazonia and the adjacent Andean forests.

The effect of geographic distance on amphibian dissimilarities could support the existence of neutral assembly rules *sensu* Hubbell (2001), especially in the Amazonian lowlands. Besides the evidences of environment filtering, we are missing the historical and evolutionary perspective of the assembly processes (Ernst et al., 2012). Historical events not taken into account could explain an important part of the regional diversity, such as processes related with geology (Hoorn et al., 2010) or climatic stability (Baselga, Gómez‐Rodríguez, & Lobo, 2012). We are still far from a comprehensive phylogeny to infer divergence times for all the taxa included in this study. However, a few studies approached these questions for particular neotropical amphibian clades. In this way, time for speciation and niche conservatism were significant in Amazonian treefrogs (Wiens et al., 2011), lungless salamanders (Kozak & Wiens, 2012), and Andean glass frogs (Hutter et al., 2013), while their effects were not more significant than current environmental variables in Terrarana (González‐Voyer, Padial, Castroviejo‐Fisher, De la Riva, & Vilà, 2011) or Central America treefrogs (Smith et al., 2007). Even more, historical interactions with other species (estimated as coexistence time) may have an effect on diversification rates (at least among treefrog clades; Wiens et al., 2011). This explains that clades with similar reproductive strategies, such as Terrarana and lungless salamanders, with different geographic origin (Andes and North America, respectively) differ in number of species in the same areas because of different colonization times. Besides the potential contribution of neutral and historical processes, our results suggest that niche‐based mechanisms play an important role at conforming the communities of amphibians at the Amazonian forests.

### 4.3 Conservation implications

Our results suggest that low primary forest holds the richest amphibian assemblage in Wisui. Land use change results in the loss of microhabitats necessary for many amphibians, reducing the total biodiversity of Amazonia through loss of primary forest and their unique species (Barlow et al., 2007), together with an increase in the abundance of particular species favored by the new conditions. Most of researchers are skeptical about the idea that regeneration will eventually reach the same diversity as mature forests (Gardner et al., 2007). Therefore, protection of primary habitats should always be a priority for conservation management. Dramatic losses in Amazonian amphibian communities occurred in sites that were monitored before major changes happened (e.g., Santa Cecilia; Duellman, 1978; Azuela; Salado) and Wisui is not an exception. Amazonian rainforests keep shrinking at alarming rates, especially in regions such as Ecuador, which lost nearly 25% of its forest in less than two decades (Peres et al., 2010). Ecological consequences of deforestation are even worse in places of high levels of endemism such as Amazonian montane and foothill forests (Myers et al., 2000). Long‐term studies have shown that relatively small reserves protecting forest remnants with enough habitat diversity might preserve species‐rich amphibian communities (Vigle, 2008). However, the resilience of mountain slope tropical rainforest assemblages with endemic species is not known. While the amphibian fauna of some localities has been properly described, there are still many areas of Amazonia whose amphibian diversity is poorly known. Many species are endemics restricted to small ranges, especially in foothill and montane areas. The knowledge about the natural history of many species of Amazonian amphibians is even scarcer. Given the high number of biotic interactions in tropical rainforests (most of them still unknown), any alteration of the network could have cascading effects on the ecosystem diversity and lead to greater loss of diversity. In summary, further local studies are necessary to understand the assembly of tropical amphibian communities and how they respond to habitat transformations due to global change, with efforts directed to know more details of their reproduction, breeding microhabitats, species interactions, and to detect other less obvious assembly processes, such as competitive exclusion.

## Conflict of interest

None declared.
