# Supplementary material for: Reproductive traits associated with species turnover of amphibians in Amazonia and its Andean slopes
Source: Ecol Evol. 2017 Mar 14;7(8):2489–500. doi: 10.1002/ece3.2862 (PMC5395459; doi:10.1002/ece3.2862)
Supplement: Supplementary file 1 [file ECE3-7-2489-s001.docx]

SUPPLEMENTARY INFORMATION

**Reproductive Traits Associated with Species Turnover of Amphibians in Amazonia and its Andean slopes**

Octavio Jiménez-Robles^1,2,5^, Juan M. Guayasamin^3,4^, Santiago R. Ron^5^ and Ignacio De la Riva^1^

^1^ Department of Biodiversity and Evolutionary Biology, Museo Nacional de Ciencias Naturales, Consejo Superior de Investigaciones Científicas, C/ José Gutiérrez Abascal, 2, 28006, Madrid, Spain.

^2^Zoology Department, Universidad de Granada, 18071, Granada, Spain.

^3^ BIÓSFERA, Laboratorio de Biología Evolutiva, Colegio de Ciencias Biológicas y Ambientales, Universidad San Francisco de Quito, Campus Cumbayá, Casilla Postal 17-1200-841, Quito 170901, Ecuador.

^4^ Centro de Investigación de la Biodiversidad y Cambio Climático, Ingeniería en Biodiversidad y Recursos Genéticos, Facultad de Ciencias de Medio Ambiente, Universidad Tecnológica Indoamérica, Calle Machala y Sabanilla, Quito, Ecuador.

^5^ Museo de Zoología, Escuela de Ciencias Biológicas, Pontificia Universidad Católica del Ecuador, Av. 12 de Octubre y Roca, Aptdo. 17-01-2184, Quito, Ecuador.

^5^ Corresponding author; email: [octavio.jimenez.robles@gmail.com](mailto:octavio.jimenez.robles@gmail.com)

Appendix S1. Description of the habitats found in Wisui Biological Station, Morona-Santiago, Ecuador.


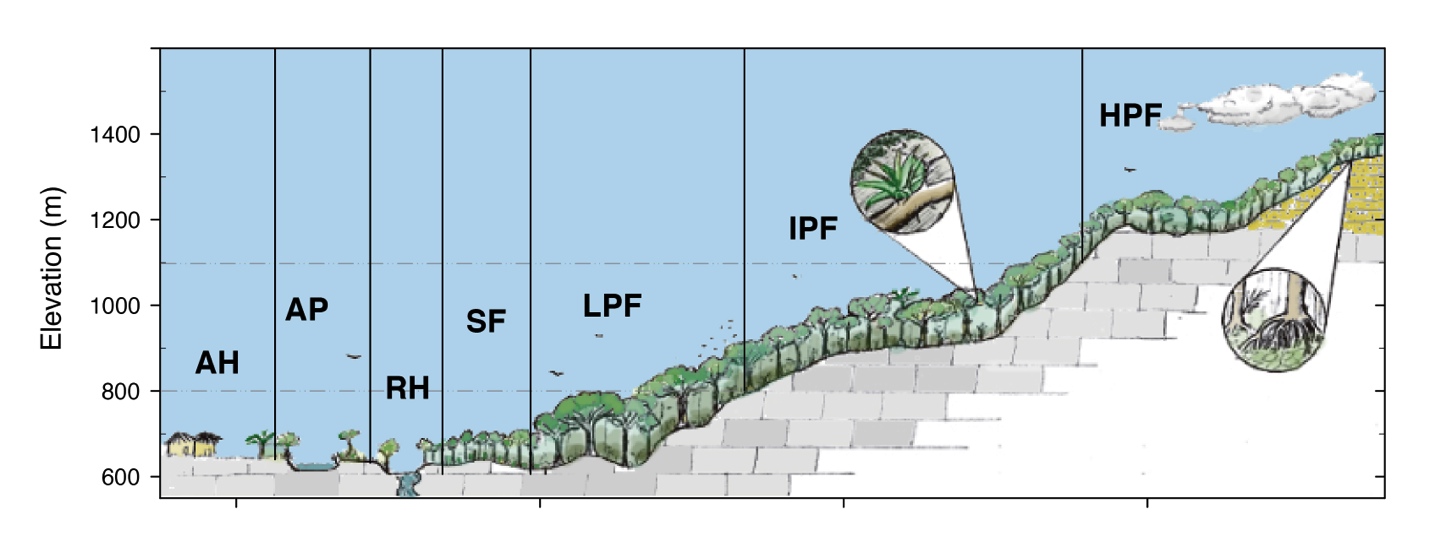


AH) Anthropogenic Habitats. Human-modified spaces, which have lost the forest structure *e.g.*: grasslands, crops, buildings, and the deforested areas of their vicinities.

AP) Artificial Pools. Habitats modified by local people for water accumulation, sometimes for aquaculture. They ranged approximately from 15 to 200 m^2^, and from 0.1 to 1 m depth.

RH) Riverine habitats. Areas adjacent to the Tayuntza and Kusutka Rivers, tributaries of the Makuma River.

SF) Secondary Forest. Habitats with arboreal cover re-grown after strong perturbations (such as felling or burning for traditional agriculture); they are featured by a less developed canopy structure, smaller trees, less epiphytes, and more vigorous ground vegetation than primary forests.

PF) Primary Forest. Habitat with no visible signs of strong anthropogenic disturbance, and trees having a wide range of widths. “Foothill Evergreen Forest of the Cordilleras del Cóndor-Kutukú” (BsPa02; Ministerio del Ambiente del Ecuador 2013). With increasing altitude and more exposed topography, the rainforest structure changes (Homeier *et al.* 2010). Therefore, we categorized primary forest into three elevation ranges:

- LPF) Low primary forest (600–800 m): habitat on limestone bedrock with moderate slopes, wide trunks and a canopy 30 m above ground. The understory is quite dense (but not that much as in SF). It has streams of 2–3 m width.
- IPF) Intermediate primary forest (800–1100 m): structurally as LPF, but on steep slopes, with streams of less than 2 m width and faster flow, and more abundant epiphyte bromeliads.
- HPF) High primary forest (1100–1400 m): with steeper slopes, neither streams nor pools, very humid air, and even more epiphyte bromeliads. At the highest elevations, the bedrock turns to sandstone, the canopy is 10–15 m above ground, with smaller crowns and leaves, and rooting is shallower. This forest, in the mountain tops, would fit within the definition of “Foothill Evergreen Forest on the Sandstone Plateau of the Cóndor-Kutukú Ranges” (BsPa04; Ministerio del Ambiente del Ecuador 2013). There is a marked increase in soil organic matter and bryophyte cover. Ferns dominate the understory.

TABLE S1. Occurrence of breeding microhabitats for amphibians in habitats of Wisui Biological Station, Morona-Santiago, Ecuador: Anthropogenic Habitats (AH), Artificial Pools (AP), Riverine Habitats (RH), Secondary Forest (SF), Low Primary Forest (LPF), Intermediate Primary Forest (IPF), and High Primary Forest (HPF). X denotes just presence, while XX denotes a remarkable abundance.

|  | **AH** | **AP** | **RH** | **SF** | **LPF** | **IPF** | **HPF** |
| --- | --- | --- | --- | --- | --- | --- | --- |
| Medium Rivers |  |  | XX |  |  |  |  |
| Wide Streams (2-3 m) |  |  |  | X | X |  |  |
| Small Streams (<2 m) |  |  |  | X | X | X |  |
| Temporary pools | X |  |  | XX | X | X |  |
| Permanent pools |  | XX |  |  |  |  |  |
| Bromeliads |  |  |  | X | X | XX | XX |
| Leaf litter |  |  |  | X | XX | XX | X |
| Moss |  |  |  | X | X | X | XX |
| Leaf-cutter ant nests | XX |  |  | X |  |  |  |


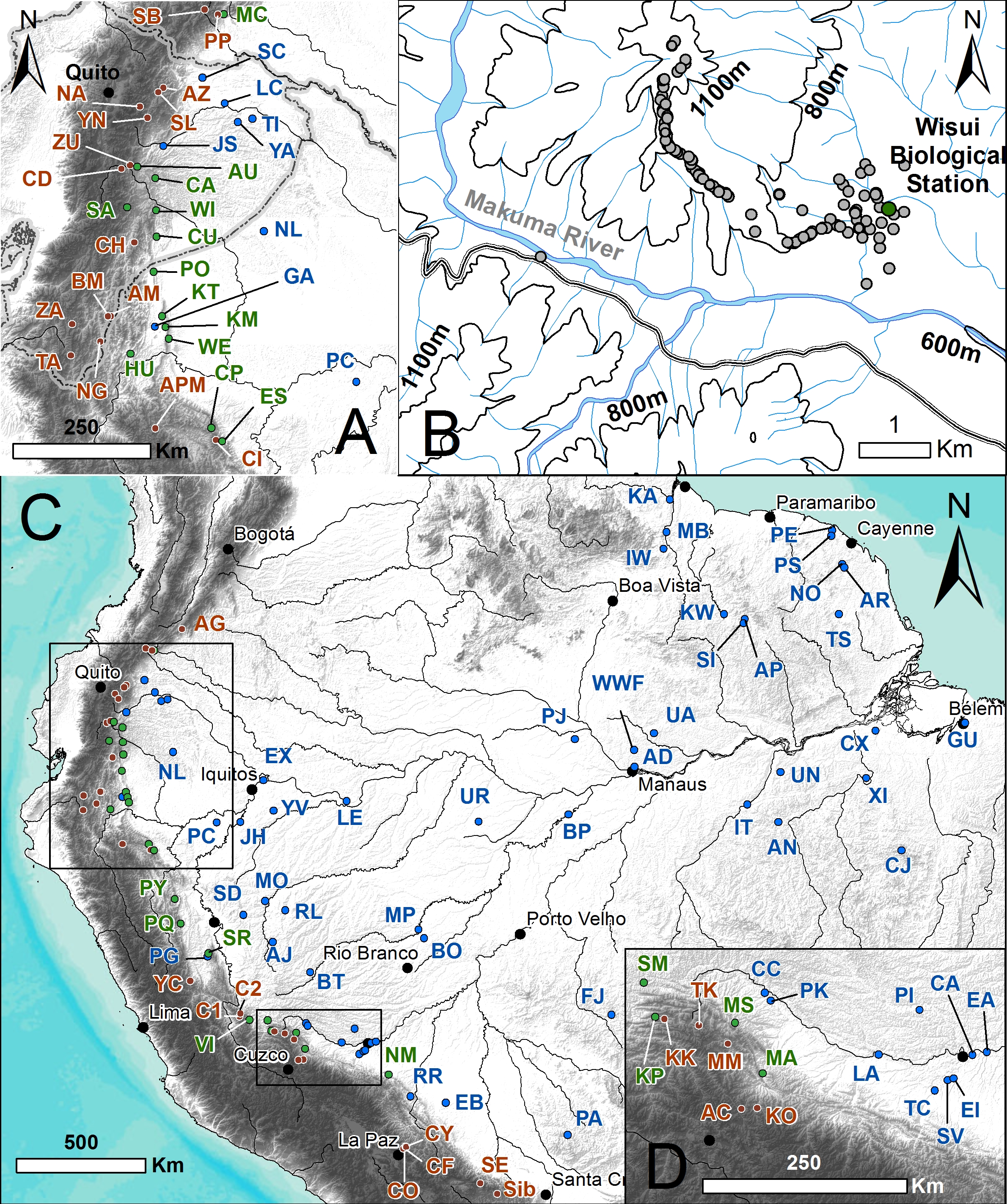


Figure S1. A) Wisui with other localities of amphibian inventories in Ecuador and Northern Peru. B) Location of the Wisui Biological Station (black circle), sampling locations (grey circles), and the trajectory of the Ebenezer-Makuma road. C) Localities of the inventories used in the biogeographic comparisons, with (D) Madre de Dios region in detail. Blue circles are lowland; green ones, foothill; and red ones, montane localities.

Table S2. Amphibians found in Wisui Biological Station with their abbreviations for Figure S2, respective reproductive modes according to Haddad and Prado (2005), the groups in which they were allocated for our analysis, and voucher specimens deposited in scientific collections. Most of the group allocations were based in literature (Duellman 1978, 2005; Hödl 1990; Zimmerman & Simberloff 1996 and other references in Table S4). In most cases RMs were conserved within large clades. Major divergences in RM of Hylidae appeared in some species, such as the three species of *Phyllomedusa, Dendropsophus bifurcus, D. brevifrons* and *D. sarayacuensis*, which lay eggs in arboreal nests hanging from vegetation above water (Crump 1974, Duellman 1978); or *Osteocephalus* aff. *fuscifacies*, which supposedly lays eggs in bromeliads (see comments below). The microhylid *Chiasmocleis antenori* also shows an independent evolution towards the oviposition in *phytotelmata*, where non-feeding tadpoles complete metamorphosis (Krügel & Richter 1995). N = eggs laid in water bodies at ground level; Veg = eggs laid on vegetation overhanging water; Phy = eggs laid on phytotelmata; FN = eggs in foam nests; Den = eggs laid on ground but larvae transported by adults to water; DD = Direct Development; ? = Unknown. Tadpole microhabitat is expressed as lentic, lotic, and phytotelm (phyt).

| **Species list** | **Abb.** | **Reproductive Modes** | | **Voucher Specimens** |
| --- | --- | --- | --- | --- |
|  |  | **Haddad & Prado 2005** | **This work** |  |
| **Gymnophiona:**  Caecilidae |  |  |  |  |
| *Caecilia sp* | A | - | ?^b^ | QCAZ 42464 |
| *Siphonops annulatus* (Mikan, 1820)^a^ | B | - | DD |  |
|  |  |  |  |  |
| **Species list** (Cont. Table S2) | **Abb.** | **Reproductive Modes** | | **Voucher Specimens** |
|  |  | **Haddad & Prado 2005** | **This work** |  |
| **Caudata:**  Plethodontidae |  |  |  |  |
| *Bolitoglossa* cf *altamazonica* (Cope 1874) | C | - | DD | QCAZ 42467 |
| *Bolitoglossa* cf *peruviana* (Boulenger, 1883) | D | - | DD | QCAZ 42479 |
| **Anura:** |  |  |  |  |
| Aromobatidae |  |  |  |  |
| *Allobates zaparo* (Silverstone, 1976) | E | 20 | Den-lotic | QCAZ 42447 |
| Bufonidae |  |  |  |  |
| *Rhaebo ecuadorensis* (Mueses-Cisneros, Cisneros-Heredia and McDiarmid, 2012) | F | 2 | N-lot./lent. | MZUTI 0390 |
| *Rhinella dapsilis* (Myers & Carvalho, 1945) | G | 2? | N-lotic | QCAZ 46369 |
| *Rhinella festae* (Peracca, 1904) | H | 2 | N-lotic^d^ | QCAZ 42430, 46351; MZUTI 0017 |
| *Rhinella margaritifera* (Laurenti, 1768) | I | 2 | N-lot./lent. | QCAZ 42422, 42426, 46349; MZUTI 0349 |
| *Rhinella marina* (Linnaeus, 1758) | J | 1 | N-lentic |  |
| *Rhinella cf roqueana* (Melin, 1941) | K | 1 | N-lentic |  |
| Centrolenidae |  |  |  |  |
| *Rulyrana flavopunctata* (Lynch & Duellman, 1973) | L | 18-25? | Veg-lotic ^e^ | QCAZ 42433, 42465-66, 42470, 46363-64; MZUTI 0042–48, 0052 |
| *Teratohyla midas* (Lynch & Duellman, 1973) | M | 25 | Veg-lotic | QCAZ 46365-66; MZUTI 0031 |
| Craugastoridae |  |  |  |  |
| *Hypodactylus nigrovittatus* (Andersson, 1945) | N | 23? | DD | QCAZ 42423, 42453, 42455, 43238, 46358-59 |
| *Noblella myrmecoides* (Lynch, 1976) | O | 23? | DD | QCAZ 40180, 40181, 46352 |
| *Oreobates quixensis* Jiménez De la Espada, 1872 | P | 23? | DD | MZUTI 0373 |
| **Species list** (Cont. Table S2) | **Abb.** | **Reproductive Modes** | | **Voucher specimens** |
|  |  | **Haddad & Prado 2005** | **This work** |  |
| *Pristimantis acuminatus* (Shreve, 1935)^a^ | Q | 27? | DD | QCAZ 42435 |
| *Pristimantis altamazonicus* (Barbour & Dunn, 1921) | R | 23-27? | DD | QCAZ 42448, 46370 |
| *Pristimantis altamnis* Elmer and Cannatella, 2008 | S | 27? | DD | QCAZ 42425, 42434, 42458-59, 46323-24, 46337; MZUTI 0037, 0352–53, 0356, 0358 |
| *Pristimantis conspicillatus* complex (Günther, | T | 23-27? | DD | clade 1: QCAZ 42424, 42474 ^c^ |
| 1858)^c^ |  |  |  | clade 2: QCAZ46355 ^c^ |
|  |  |  |  | non-assigned clade: QCAZ 4227-29, 42431, 42437, 42439, 42441, 42450, 42454, 42456-57, 42471, 42476, 43237, 46354; MZUTI 0010, 0035 |
| *Pristimantis croceoinguinis* (Lynch, 1968) | U | 27? | DD | QCAZ 42461, 46336; MZUTI 0354 |
| *Pristimantis diadematus* (Jiménez De la Espada, 1875) | V | 27? | DD | QCAZ 42440, 42445, 46362 |
| *Pristimantis katoptroides* (Flores, 1988) | W | 27? | DD | QCAZ 46360 |
| *Pristimantis lanthanites* (Lynch, 1975) | X | 23-27? | DD | QCAZ 46348; MZUTI 0359 |
| *Pristimantis martiae* (Lynch, 1974) | Y | 23? | DD | QCAZ 46350-53 |
| *Pristimantis rubicundus* (Jiménez De la Espada, 1875) | Z | 23? | DD | QCAZ 46361 |
| *Pristimantis* sp 1 | a | 27? | DD | QCAZ 42469 |
| *Pristimantis* sp 2 | b | 23? | DD | QCAZ 43236 |
| *Pristimantis trachyblepharis* (Boulenger, 1918) | c | 27? | DD | QCAZ 42436, 42451-52, 42472; MZUTI 0040 |
| *Strabomantis sulcatus* (Cope, 1874) | d | 23? | DD | QCAZ 42444 |
|  |  |  |  |  |
| **Species list** (Cont. Table S2) | **Abb.** | **Reproductive Modes** | | **Voucher specimens** |
|  |  | **Haddad & Prado 2005** | **This work** |  |
| Dendrobatidae |  |  |  |  |
| *Ameerega parvula* (Boulenger, 1882) | e | 20 | Den-lotic | QCAZ 48949; MZUTI 0013, 0049, 0378 |
| *Hyloxalus cf cevallosi* (Rivero, 1991)^a^ | f | 20 | Den-lotic | MZUTI 0380–82 |
| *Ranitomeya variabilis* Zimmerman & Zimmerman, 1998 | g | 20 | Den-phyt ^f^ | QCAZ 43235; MZUTI 0018 |
| Hemiphractidae |  |  |  |  |
| *Hemiphractus proboscideus* (Jiménez De la Espada, 1870) | h | 37 | DD |  |
| Hylidae |  |  |  |  |
| *Dendropsophus bifurcus* (Andersson, 1946) | i | 24 | Veg-lentic | QCAZ 42477, 46340-41; MZUTI 0004–05, 0022, 0365-70 |
| *Dendropsophus brevifrons* (Duellman & Crump, 1974) | j | 24 | Veg-lentic | QCAZ 46368 |
| *Dendropsophus marmoratus* (Laurenti, 1768) | k | 1 | N-lentic | MZUTI 0014, 0028, 0038, 0350, 0375 |
| *Dendropsophus rhodopeplus* (Günther, 1858) | l | 1 | N-lentic | QCAZ 46343-44; MZUTI 0003, 0009, 0011, 0024 |
| *Dendropsophus sarayacuensis* (Shreve, 1935) | m | 24 | Veg-lentic | QCAZ 46342; MZUTI 0002, 0006–08, 0012, 0023 |
| *Hypsiboas boans* (Linnaeus, 1758) | n | 4 | N |  |
| *Hypsiboas cinerascens* (Spix, 1824) | o | 1-2 | N | QCAZ 46371 |
| *Hypsiboas lanciformis* Cope, 1871 | p | 1 | N-lentic | QCAZ 42432, 42443, 46338-39 |
| *Osteocephalus* aff. *fuscifacies* Jungfer, Ron, Almendáriz & Seipp 2000 | q | 6 | Phy^g^ | QCAZ 42473 |
| *Osteocephalus mutabor* Jungfer & Hödl, 2002 | r | 1 | N-lentic | QCAZ 46367; MZUTI 0026, 0032–34, 0041, 0379 |
| *Osteocephalus yasuni* Ron & Pramuk 1999 | s | 1 | N-lentic | QCAZ 42449; MZUTI 0377 |
| **Species list** (Cont. Table S2) | **Abb.** | **Reproductive Modes** | | **Voucher specimens** |
|  |  | **Haddad & Prado 2005** | **This work** |  |
|  |  |  |  |  |
| *Scinax garbei* (Miranda-Ribeiro 1926) | t | 24 | N-lentic | QCAZ 42463, 46347; MZUTI 0371–72 |
| Leptodactylidae |  |  |  |  |
| *Leptodactylus knudseni* Heyer 1972 | u | 29 | FN-lentic | QCAZ 48326 |
| *Leptodactylus mystaceus* (Spix 1824) | v | 29 | FN-lentic | QCAZ 42442 |
| *Leptodactylus rhodomystax* (Boulenger 1884)^a^ | w | 11 | FN-lentic | MZUTI 0019, 0029 |
| *Leptodactylus wagneri* (Peters 1862) | x | 11 | FN-lentic | QCAZ 42460, 46356; MZUTI 0016 |
| *Lithodytes lineatus* (Schneider 1799) | y | 11? | FN-lentic^h^ | QCAZ 48327; MZUTI 0021, 0027, 0039, 0051, 00348, 00374, 00383 |
| Microhylidae |  |  |  |  |
| *Chiasmocleis antenori* (Walker 1973) | z | 8 | Phy | QCAZ 42462, 42468 |
| *Chiasmocleis bassleri* Dunn 1949^a^ | AA | 1 | N-lentic | QCAZ 42478, 42480; MZUTI 0376 |
| Phyllomedusidae |  |  |  |  |
| *Phyllomedusa tarsius* (Cope 1868) | AB | 24 | Veg-lentic | QCAZ 46345; MZUTI 0001, 0015, 0025, 0389 |
| *Phyllomedusa tomopterna* (Cope 1868) | AC | 24 | Veg-lentic | QCAZ 46346; MZUTI 0036 |
| *Phyllomedusa vaillanti* Boulenger 1882 | AD |  | Veg-lentic | QCAZ 46357 |
|  |  |  |  |  |

^a^ Only caught opportunistically

^b^ We could not assign *Caecilia* sp. to any category, due to the high diversity of reproductive modes inside its genus (Duellman & Trueb 1986).

^c^ What we call *P. conspicillatus* is actually two undescribed cryptic lineages (T. Camacho-Badani and S. Ron, pers. comm.) which we could not distinguish during the fieldwork in Wisui.

^d^ The only datum about reproduction in *Rhinella festae* is that a gravid female contained “relatively few, large (1.8 mm), unpigmented eggs” (Trueb 1971). Pereyra *et al.* (2015) did not discard the existence of direct development in members of the *Rhinella acrolopha* group (former genus *Rhampophryne*), but without further evidence. Conservatively, we opted to assume that *R. festae* lay eggs on waterbodies, possibly in lotic environments, but future observations on this group might provide evidence of a different reproductive mode.

^e^ Clutches of *Rulyrana flavopunctata* in Wisui, were observed on rock surfaces above stream water (further details in Jiménez-Robles *et al.* 2015)

^f^ *Ranitomeya variabilis* tadpoles, after hatching are carried by adults to phytotelm bromeliads where they develop (Brown *et al.* 2012).

^g^ This *Osteocephalus* proved to belong to an undescribed lineage within the *O. planiceps* group sensu Jungfer *et al*. (2013) according to phylogenetic analyses using the material collected (QCAZ 42473; S. Ron, pers. comm.). Because phytotelm breeding is considered a putative synapomorphy of this group and we found one male (QCAZ 42473) calling from a water-containing bromeliad, we suppose it breeds in this microhabitat.

^h^ The little we know about *Lithodytes lineatus* reproduction is that its tadpoles have no dark pigmentation and in nature have been only found in the deeper and flooded chambers inside leaf cutter ants nests (genus *Atta*), probably a consequence of a symbiotic coevolution (Schlüter *et al.* 2009)

Table S3. Localities used in the biogeographical analysis of anuran inventories, with the data source (fully detailed in Appendix S2) and their number of species, unique species (Excl), and unidentified species (UnID), and elevation range.

| **Code** | **Locality** | **Country** | **Spp^a^** | **Excl** | **UnID** | **Elevation (m asl)** | **Source** |
| --- | --- | --- | --- | --- | --- | --- | --- |
| SC | Santa Cecilia | Ecuador | 92 | 0 | 0 | 340 | Crump 1974, Duellman 1978 |
| LC | Limoncocha | Ecuador | 66 | 1 | 0 | 220 | Duellman & Thomas 1996 |
| YA | Yasuní | Ecuador | 82 | 0 | 0 | 230-600 | Ron *et al.* 2015 |
| TI | Tiputini | Ecuador | 112 | 2 | 1 | 190-270 | Cisneros-Heredia 2006, Guayasamin *et al.* 2006, McCracken *et al.* 2007 |
| JS | Jatún Sacha | Ecuador | 81 | 3 | 2 | 400-450 | Vigle 2008 |
| NL | Northern Loreto^b^ | Peru | 67 | 1 | 2 | 175-340 | Duellman & Mendelson 1995 |
| GA | Puerto Galilea-La Poza | Peru | 60 | 6 | 7 | 180 | MVZ & USNM collections by J.E. Cadle in vertnet.org |
| PC | Pacaya-Samiría^b^ | Peru | 56 | 4 | 4 | 115-120 | Upton 2015 |
| JH | Jenaro Herrera | Peru | 74 | 2 | 2 | 130 | Guerrero *et al.* 2010 |
| EX | Explorama | Peru | 65 | 1 | 1 | 100 | Duellman & Rodríguez, 1991-1994 in Duellman & Thomas 1984 |
| YV | Yavarí^b^ | Peru | 91 | 12 | 10 | 115–150 | Rodríguez & Knell, Perez *et al* .2005 |
| LE | Leticia | Colombia | 96 | 8 | 5 | 83 | Lynch 2005 |
| RL | R. da Liberdade | Brazil | 83 | 6 | 6 | 204 | Bernarde *et al.* 2011 |
| MO | Lower Moa River | Brazil | 49 | 2 | 2 | 194 | Miranda *et al.* 2015 |
| SD | Serra do Divisor^b^ | Brazil | 120 | 3 | 11 | 220-320 | Souza 2009 |
| AJ | Alto Juruá^b^ | Brazil | 110 | 0 | 8 | 230-300 | Souza 2009 |
| PG | Panguana | Peru | 72 | 6 | 5 | 200 | Toft & Duellman 1979, Schlüter 1984 in Duellman & Thomas 1984, Schlüter *et al.* 2004, Rödder & Schlüter 2009 |
| BT | Balta | Peru | 55 | 0 | 0 | 300 | Duellman & Thomas 1996 |
| CC | Cocha Cashu | Peru | 78 | 1 | 1 | 300-400 | Rodríguez & Cadle 1990, Rodríguez 1992 in von May *et al.* 2008 |
| **Code** | **Locality** | **Country** | **Spp^a^** | **Excl** | **UnID** | **Elevation (m asl)** | **Source** |
| PK | Pakitza | Peru | 63 | 0 | 0 | 329 | Morales & McDiarmid 1996 in von May *et al.* 2008 |
| LA | Los Amigos | Peru | 81 | 1 | 2 | 244 | von May *et al.* 2008 |
| PI | Las Piedras | Peru | 58 | 0 | 0 | 279 | von May *et al.* 2008 |
| TC | Tambopata R. C. | Peru | 74 | 1 | 1 | 215 | Doan & Arizabal 2002, Duellman 2005 |
| SV | Sachavacayoc | Peru | 56 | 0 | 0 | 215 | Doan & Arizabal 2002, Duellman 2005 |
| EI | Explorer's Inn | Peru | 75 | 1 | 1 | 209 | Doan & Arizabal 2004, Duellman 2005 |
| CA | Cusco Amazónico | Peru | 67 | 0 | 0 | 200 | Duellman 2005 |
| EA | EcoAmazonía | Peru | 51 | 0 | 0 | 200 | Doan & Arizabal 2002, Duellman 2005 |
| RR | Rurrenabaque & Chalalánb | Bolivia | 54 | 0 | 0 | 200-400 | Reichle 2006; records in vertnet.org |
| EB | Beni Biological Station | Bolivia | 33 | 3 | 2 | 220 | Middendorf & Reynolds 2000 |
| PA | Puerto Almacén | Bolivia | 42 | 2 | 0 | 300 | De la Riva 1993 |
| FJ | Facenda Jaburi | Brazil | 48 | 4 | 4 | 280 | Bernarde 2007 |
| BO | Boca do Acre^b^ | Brazil | 58 | 0 | 0 | 100-150 | França & Venâncio 2010 |
| MP | Middle Purus^b^ | Brazil | 58 | 4 | 1 | 100-130 | Ramalho *et al.* 2016 |
| UR | Urucu^b^ | Brazil | 55 | 5 | 5 | 90 | Prudente *et al.* 2013 |
| PJ | P.N. do Jau^b^ | Brazil | 43 | 7 | 6 | 25 | Neckel-Oliveira & Gordo 2004 |
| BP | Baixo Purus^b^ | Brazil | 74 | 4 | 3 | 40 | Waldez *et al.* 2013 |
| WWF | INPA-WWF | Brazil | 44 | 2 | 1 | 25 | Zimmerman & Rodrigues 1990 |
| AD | R.F. A. Ducke | Brazil | 52 | 1 | 1 | 100 | Lima *et al.* 2006 |
| UA | Uatuma | Brazil | 59 | 5 | 5 | 52-195 | Condrati 2009 |
| IT | Itaituba | Brazil | 39 | 2 | 0 | 24 | USNM records in vertnet.org |
| AN | R. de Anfrísio^b^ | Brazil | 57 | 5 | 4 | 182 | Barros *et al.* 2014 |
| UN | Curua Una | Brazil | 37 | 2 | 5 | 96 | Caldwell & Araújo |
| XI | Rio Xingu | Brazil | 40 | 3 | 6 | 110 | Caldwell & Araújo |
| CJ | Carajás^b^ | Brazil | 70 | 12 | 2 | 250-800 | Pinheiro *et al.* 2012 |
| CX | Caxiuanã | Brazil | 40 | 0 | 0 | 43 | Estupiñán *et al.* 2002 |
| GU | Guamá | Brazil | 40 | 4 | 2 | 0-12 | Crump 1971 |
| KA | Kartabo | Guyana | 43 | 2 | 0 | 100 | Beebe 1946 in Cole *et al.* 2013 |
| **Code** | **Locality** | **Country** | **Spp^a^** | **Excl** | **UnID** | **Elevation (m asl)** | **Source** |
| MB | Mabura | Guyana | 42 | 4 | 3 | 93 | Ernst *et al*. 2005 |
| IW | Iwokrama | Guyana | 56 | 16 | 15 | 70-224 | Donnelly *et al.* 2005 |
| KW | Kwamalasamutu | Suriname | 38 | 1 | 2 | 205 | Ouboter *et al.* 2011; Fouquet *et al.* 2015 |
| AP | Apalagadi | Suriname | 55 | 2 | 2 | 320 | Fouquet *et al.* 2015 |
| SI | Sipaliwini | Suriname | 59 | 8 | 7 | 280 | Fouquet *et al.* 2015 |
| PE | Piste of St. Ellie | F. Guiana | 34 | 0 | 2 | 45 | Born & Gaucher 2001 |
| PS | Petit Saut | French Guiana | 38 | 1 | 2 | 67 | Hoogmoed & Avila-Pires, 1991; Duellman 1997 in Born & Gaucher 2001 |
| NO | Nouragues | F.Guiana | 54 | 1 | 2 | 40-400 | Born & Gaucher 2001 |
| AR | Arataye | F. Guiana | 65 | 2 | 4 | 93 | Born & Gaucher 2001 |
| TS | Trois Sauts | F. Guiana | 64 | 8 | 7 | 161 | Lescure 1982 in Born & Gaucher 2001 |
| AG | Alto de Gabinete | Colombia | 36 | 17 | 7 | 1000–2450 | Suárez-Mayorga 1999, Mueses-Cisneros 2005 |
| SB | Sibundoy | Colombia | 32 | 14 | 5 | 1950–2750 | Mueses-Cisneros 2005 |
| MC | Mocoa | Colombia | 32 | 6 | 6 | 500–700 | Betancourth-Cundar & Gutiérrez-Zamora 2010 |
| PP | 10.3km W El Pepino | Colombia | 20 | 2 | 2 | 1430 | KUH & MCZ collections by W. Duellman in vertnet.org |
| AZ | Azuela | Ecuador | 30 | 4 | 0 | 1740 | KUH, MCZ and USNM collections in vertnet.org |
| SL | Salado | Ecuador | 24 | 1 | 0 | 1410 | KUH, MCZ and USNM collections in vertnet.org |
| NA | Napo | Ecuador | 26 | 7 | 0 | 1850–2850 | Lynch & Duellman 1980; Mueses-Cisneros 2005 |
| YN | Yanayacu | Ecuador | 18 | 5 | 2 | 2100–2300 | Guayasamin & Funk 2009 |
| CD | Candelaria | Ecuador | 25 | 12 | 7 | 1800–3300 | Yáñez-Muñoz & Reyes-Puig 2008; Reyes-Puig |
| ZU | Zúñac | Ecuador | 29 | 5 | 1 | 1400–2200 | Yáñez-Muñoz & Reyes-Puig 2008; Reyes-Puig *et al.* 2013; 2015 |
| AU | Anzu | Ecuador | 32 | 6 | 6 | 1050–1300 | Yáñez-Muñoz & Reyes-Puig 2008 |
| CN | Canelos | Ecuador | 41 | 0 | 1 | 503 | Ortega-Andrade 2010 |
|  |  |  |  |  |  |  |  |
| **Code** | **Locality** | **Country** | **Spp^a^** | **Excl** | **UnID** | **Elevation (m asl)** | **Source** |
| SA | Sangay^b^ | Ecuador | 65 | 9 | 4 | 700–2000 | Brito & Almendáriz 2013; Harvey *et al*. 2013; Batallas & Brito 2014 |
| WI | Wisui | Ecuador | 56 | 4 | 4 | 640-1360 | this study |
| CU | Cusuimi | Ecuador | 61 | 4 | 5 | 600 | Malkin 1971 in Ortega-Andrade 2010 |
| CH | Chiguaza | Ecuador | 26 | 5 | 1 | 1075–1975 | Duellman & Lynch 1988b |
| PO | Pongo Chinin | Peru | 33 | 2 | 4 | 365–720 | Catenazzi & Venegas 2012 |
| KT | Katerpiza | Peru | 34 | 2 | 7 | 300–1340 | Catenazzi & Venegas 2012 |
| KM | Kampankis | Peru | 31 | 1 | 0 | 325–1020 | Catenazzi & Venegas 2012 |
| WE | Wee | Peru | 37 | 1 | 4 | 310–1435 | Catenazzi & Venegas 2012 |
| **HU** | Huampami | Peru | 46 | 0 | 0 | 470 | USNM, MCZ, MVZ collections of J. E. Cadle in vertnet.org; Páez-Vacas *et al*. 2010 |
| AM | Alto Machinaza 1850–2400 m | Ecuador | 36 | 19 | 15 | 1850–2400 | Almendáriz *et al.* 2014 |
| BM | Alto Machinaza | Ecuador | 51 | 12 | 9 | 1300-1850 | Almendáriz *et al.* 2014 |
| NG | Miazi & Alto Nangaritza | Ecuador | 38 | 8 | 6 | 950-1850 | Almendáriz *et al.* 1997; Guayasamín *et al.* 2011; Almendáriz *et al.* 2014 |
| ZA | Abra de Zamora | Ecuador | 12 | 7 | 0 | 2850 | KUH & MCZ collections by W. Duellman in vertnet.org |
| TA | Tapichalaca | Ecuador | 24 | 10 | 5 | 1850-2850 | Ramírez et al 2009 |
| APM | Abra de Pardo Miguel | Peru | 24 | 7 | 0 | 300–2000 | Duellman & Wiens 1993; Duellman 2004; Catenazzi & Venegas 2012; vertnet.org |
| CP | Alto Cahuapanas | Peru | 25 | 3 | 6 | 1000–1350 | Venegas *et al.* 2014 |
| CI | Alto Cachiyacu | Peru | 18 | 3 | 3 | 800–1950 | Venegas *et al.* 2014 |
| ES | Escalera Lowlands: Mina de Sal & Alto Cachiyacu Base | Peru | 49 | 5 | 3 | 300–800 | Venegas *et al.* 2014 |
| PY | Pauya | Peru | 35 | 8 | 10 | 360–1440 | Rodríguez *et al*. 2001 |
| PQ | Pisqui | Peru | 30 | 3 | 7 | 200–1220 | Rodríguez *et al.* 2001 |
| SR | Sira | Peru | 18 | 3 | 0 | 690–1280 | Duellman & Toft 1979 |
| YC | Yanachaga Cloud Forests | Peru | 41 | 32 | 5 | 2000–3400 | Angulo *et al.* 2015 |
| **Code** | **Locality** | **Country** | **Spp^a^** | **Excl** | **UnID** | **Elevation (m asl)** | **Source** |
| C1 | Camp 1 RAP Vilcabamba | Peru | 3 | 3 | 3 | 3350 | Rodríguez 2001 |
| C2 | Camp 2 RAP Vilcabamba | Peru | 11 | 8 | 8 | 2050 | Rodríguez 2001 |
| VI | Vilcabamba Ridge Camp | Peru | 29 | 7 | 7 | 500–1200 | Rodríguez 2001 |
| SM | San Martín 3 | Peru | 40 | 0 | 0 | 474 | Reynolds & Morales 1997; vertnet; Duellman & Lehr 2009 |
| KP | Kapiromashi | Peru | 15 | 1 | 1 | 650–1200 | Rodríguez & Catenazzi 2004 |
| KK | Katarompanaki | Peru | 11 | 3 | 4 | 1300–2000 | Rodríguez & Catenazzi 2004 |
| TK | Tinkanari | Peru | 10 | 5 | 5 | 1800–2600 | Rodríguez & Catenazzi 2004 |
| MS | Manu Submontane^b^ | Peru | 59 | 1 | 0 | 500–1000 | Catenazzi *et al*. 2013 |
| MM | Manu Cloud forest^b^ | Peru | 43 | 10 | 3 | 1000–3600 | Catenazzi *et al.* 2013 |
| MA | Manu Learning C. | Peru | 61 | 7 | 4 | 450-750 | Whitworth & Villacampa-Ortega 2015 |
| KO | Kosñipata | Peru | 23 | 3 | 2 | 1700 | KUH & MCZ collections by W. Duellman in vertnet.org |
| AC | Abra de Acanacu | Peru | 7 | 3 | 1 | 3250 | KUH collections in vertnet.org |
| NM | Northern Madidi^b^ | Bolivia | 46 | 3 | 2 | 200-800 | Cortez Fernandez 2005 |
| CO | Cotapata Páramo | Bolivia | 6 | 3 | 1 | 3200–3900 | Cortez Fernandez 2006 |
| CF | Cotapata Cloud forests | Bolivia | 7 | 2 | 1 | 2350–3250 | Cortez Fernandez 2006 |
| CY | Cotapata Yungas | Bolivia | 17 | 2 | 1 | 1190–2155 | Cortez Fernandez 2006 |
| SE | Sehuencas | Bolivia | 12 | 3 | 0 | 2100–2300 | Köhler 2000; Reichle 2006 |
| Sib | La Siberia | Bolivia | 16 | 5 | 0 | 2450–2650 | Köhler 2000; Reichle 2006 |
|  |  | N |  |  |  |  |  |
|  | **Lowlands** | 57 | 466 | 171 | 141 | 0–400 |  |
|  | **Foothills** | 22 | 292 | 76 | 68 | 400–1200 |  |
|  | **Montane** | 30 | 394 | 220 | 96 | 1200–3900 |  |
|  | Total | 109 | 897 | 467 | 302 |  |  |

^a^ Synonymies were corrected according to current systematic publications (as in Frost 2017). Some species complexes were treated as a single species due to the identification problems they involve: *Rhinella margaritifera* (*“Bufo typhonius”*), *Scinax ruber* (Fouquet *et al.* 2007), *Adenomera* (Angulo *et al.* 2003, Angulo & Reichle 2008), *Pristimantis ockendeni/altamnis/achuar/kichwarum* (Elmer & Cannatella 2008), *Leptodactylus mystaceus/didymus* (Heyer *et al.* 1996), *Rhaebo guttatus / glaberrimus/ecuadorensis* (Mueses-Cisneros *et al.* 2012), *Dendropsophus minutus/delarivai/xapurensis* (Köhler & Lötters 2001, Faivovich *et al.* 2005), *Hypsiboas calcaratus/fasciatus/almendarizae/maculateralis* (Funk *et al.* 2011, Caminer & Ron 2014), and the genus *Ranitomeya* (Brown *et al.* 2011). Therefore, richness is not the real known species number of those sites, as in many inventories there were more than one species in the same conflictive species complex. All these references are detailed in Table S5.

^b^ Inventories involving more than one sampling location in larger areas than the others. Therefore, observed richness is more than we would expect from a single locality.


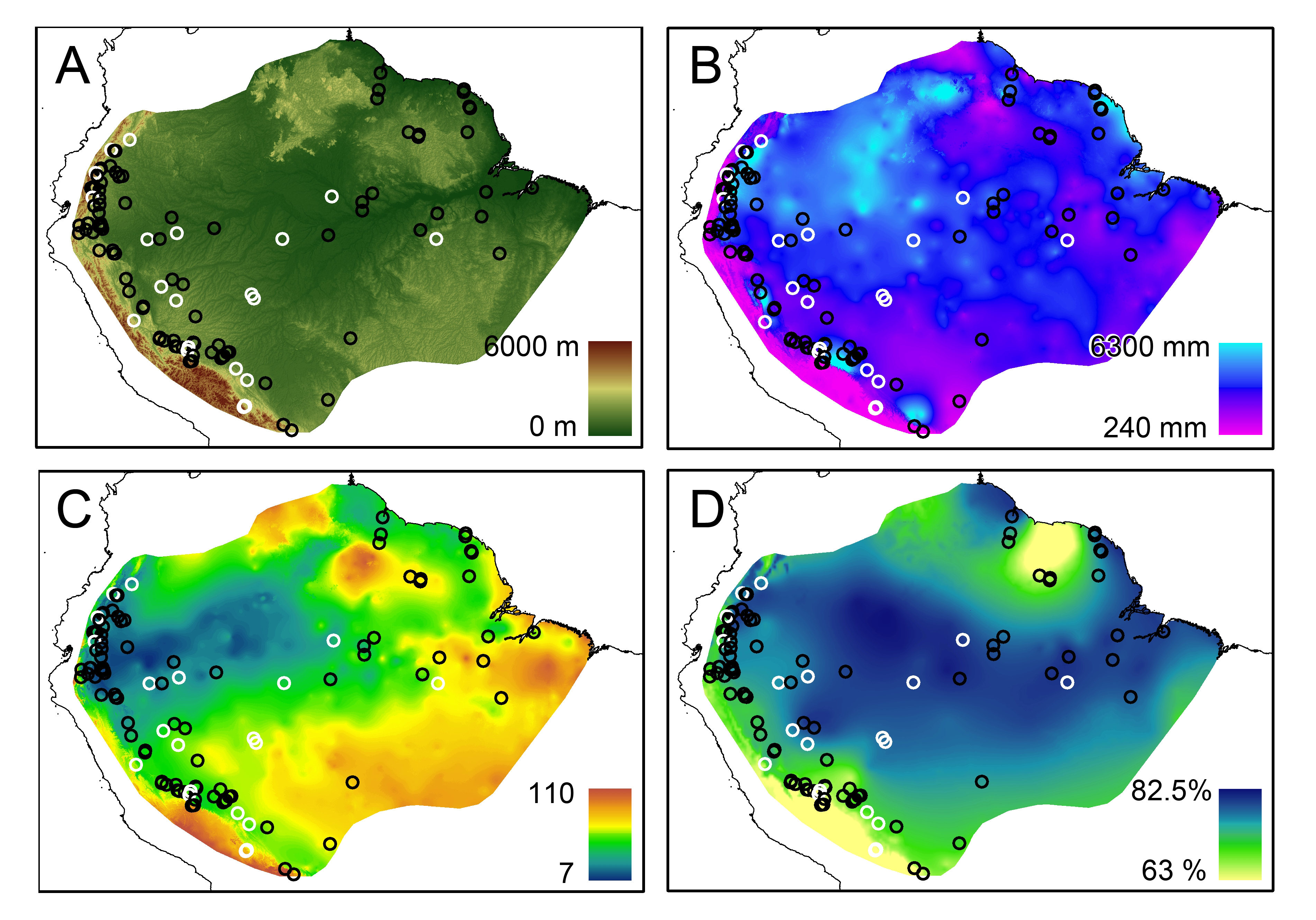


Figure S2. Geographic distribution of the amphibian inventories that we used in our analyses, overlaid on top of different environmental variables: A) elevation, B) total precipitation, C) precipitation seasonality (coefficient of variation) (from WorldClim, Hijmans *et al.,* 2005), and D) mean annual relative humidity (averaged from New *et al.,* 2002 layers). White circles are those localities excluded from the total species richness model. Black circles are the rest of localities.


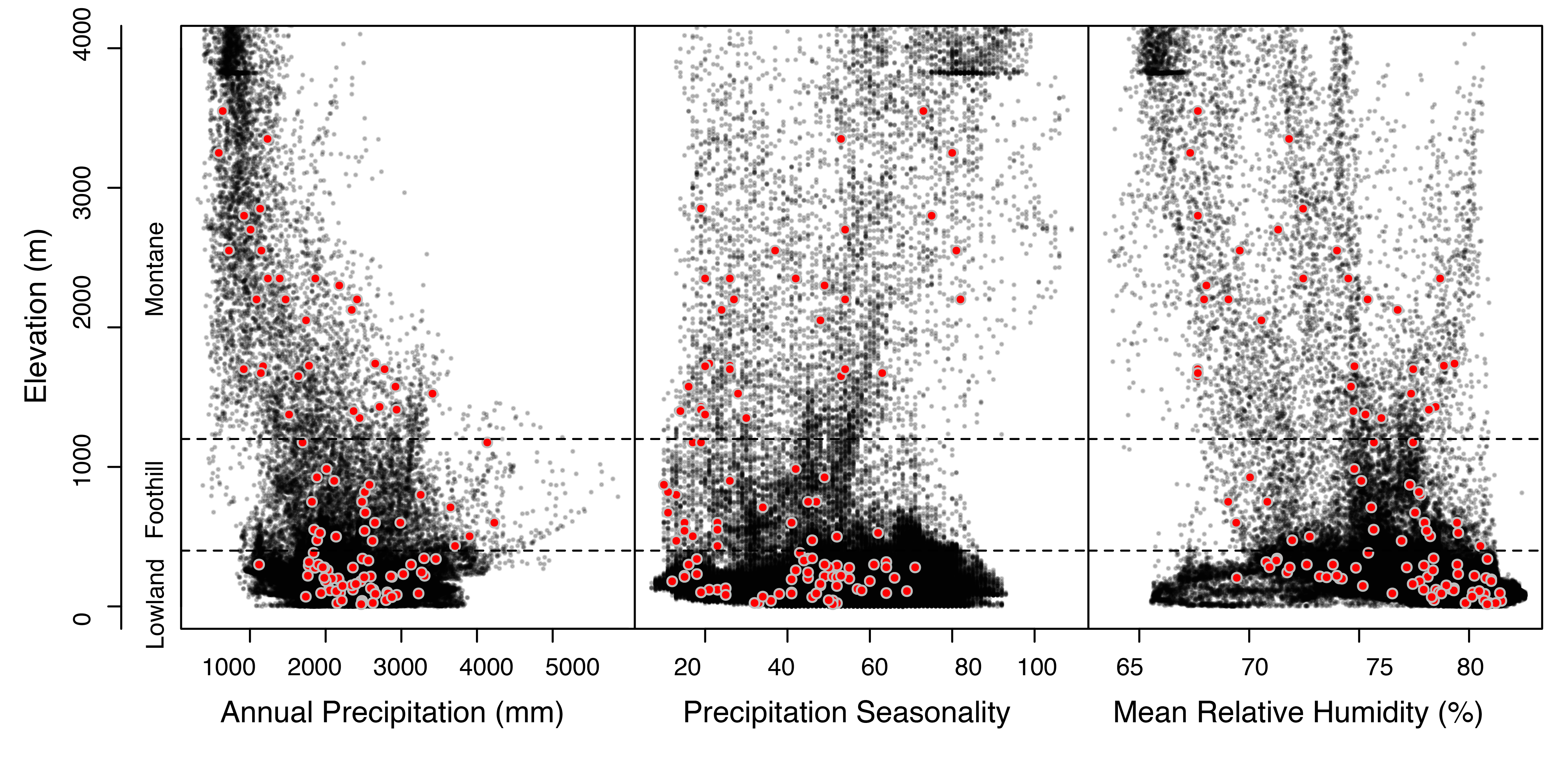


Figure S3. Elevational variation of total precipitation, precipitation seasonality, and mean annual relative humidity in 100,000 random points within the area that includes Amazonia and its Andean slopes (the same polygon we used in Figures 4 and S2). Apart from evergreen forests, there are some patches of different ecosystems included, such as the Tepuyes, the Guianan Savannas, Beni Savannas, Chiquitano forest, Cerrado, Páramo and Puna. The evergreen forest localities we use in our analyses (red circles) are representative of the ecological conditions found in the evergreen forests of Amazonia and its Andean slopes.

Appendix S2. List of full bibliographic references used in the compilation of reproductive modes of the amphibians in Figure 1, Tables S2 and S3 and the Amazonian anuran inventories in Figure S1 and Table S3.

Almendáriz, A. (1997) Herpetofauna of Miazi. *The Cordillera del Condor region of Ecuador and Peru: A biological assessment.* (ed. by T.S. Schulenberg and K. Awbrey), p. 82. Conservation International.

Almendáriz, A., Simmons, J., Brito, J. & Vaca-Guerrero, J. (2014) Overview of the herpetofauna of the unexplored Cordillera del Cóndor of Ecuador. *Amphibian & Reptile Conservation*, **8**, 45-64.

Angulo, A., Cocroft, R.B. & Reichle, S. (2003) Species identity in the genus *Adenomera* (Anura: Leptodactylidae) in southeastern Peru. *Herpetologica*, **59**, 490-504.

Angulo, A., May, R.v., Icochea, J., Chaparro, J.C., Hedges, S.B., Lehr, E., López, D.G., Lundberg, M. & Moravec, J. (2016) Anfibios del bosque de nubes y su zona de amortiguamiento. Parque Nacional Yanachaga-Chemillén, Pasco, Perú. *Field Museum Rapid Color Guides*, **738**, 1–7.

Barros, F.B., Pereira, H.M. & Vicente, L. (2014) Anfíbios anuros da Reserva Extrativista Riozinho do Anfrísio (Pará, Brasil). *Revista Gaia Scientia*, **8**, 156-173.

Batallas, R. & Brito, M. (2014) Nueva especie de rana del género *Pristimantis* del grupo *lacrimosus* (Amphibia: Craugastoridae) del Parque Nacional Sangay, Ecuador. *Papéis Avulsos de Zoologia*, **54**, 51-62.

Bernarde, P.S. (2007) Ambientes e temporada de vocalização da anurofauna no Município de Espigão do Oeste, Rondônia, Sudoeste da Amazônia - Brasil (Amphibia: Anura). *Biota Neotropica*, **7**, 87-92.

Bernarde, P.S.r., Machado, R.A.n. & Turci, L.C.B. (2011) Herpetofauna of Igarapé Esperança area in the Reserva Extrativista Riozinho da Liberdade, Acre, Brazil. *Biota Neotropica*, **11**, 117-144.

Betancourth-Cundar, M. & Gutiérrez, A. (2010) Aspectos ecológicos de la herpetofauna del Centro Experimental Amazónico, Putumayo, Colombia. *Ecotrópicos*, **23**, 61-78.

Brown, J.L., Twomey, E., Amezquita, A., Souza, M., Caldwell, J.P., Lötters, S., von May, R., Melo-Sampaio, P.R., Mejia-Vargas, D. & Perez-Pena, P. (2011) A taxonomic revision of the Neotropical poison frog genus *Ranitomeya* (Amphibia: Dendrobatidae). *Zootaxa*, **3083**, 1-120.

Caldwell, J.P. & de Araújo, M.C. (2005) *Amphibian faunas of two eastern Amazonian rainforest sites in Pará, Brazil*. Sam Noble Oklahoma Museum of Natural History.

Caminer, M.A. & Ron, S.R. (2014) Systematics of treefrogs of the *Hypsiboas calcaratus* and *Hypsiboas fasciatus* species complex (Anura, Hylidae) with the description of four new species. *ZooKeys*, **370**, 1-68.

Catenazzi, A. & Venegas, P.J. (2012) Amphibians and reptiles. *Perú: Cerros de Kampankis. Rapid Biological and Social Inventories Report 24* (ed. by N. Pitman, E.R. Inzunza, D. Alvira, C. Vriesendorp, D.K. Moskovits, Á.D. Campo, T. Wachter, D.F. Stotz, S.N. Sesén, E.T. Cerrón and R.C. Smith), pp. 260–271. The Field Museum, Chicago.

Catenazzi, A., Lehr, E. & May, R.v. (2013) The amphibians and reptiles of Manu National Park and its buffer zone, Amazon basin and eastern slopes of the Andes, Peru. *Biota Neotropica*, **13**, 269-283.

Cisneros-Heredia, D.F. (2006) *La herpetofauna de la Estación de Biodiversidad Tiputini, Ecuador.* Universidad San Francisco de Quito, Quito.

Cole, C.J., Townsend, C.R., Reynolds, R.P., MacCulloch, R.D. & Lathrop, A. (2013) Amphibians and reptiles of Guyana, South America: illustrated keys, annotated species accounts, and a biogeographic synopsis. *Proceedings of the Biological Society of Washington*, **125**, 317-578.

Cortez Fernandez, C. (2005) Herpetofauna de la zona norte del Parque Nacional y Area Natural de Manejo Integrado Madidi (PNANMI-Madidi). *Ecología en Bolivia*, **40**, 10-26.

Cortez Fernandez, C. (2006) Variación altitudinal de la riqueza y abundancia relativa de los anuros del Parque Nacional y Área Natural de Manejo Integrado Cotapata. *Ecología en Bolivia*, **41**, 46-64.

Crump, M.L. (1971) Quantitative analysis of the ecological distribution of a tropical herpetofauna. *Occasional Papers of the Natural History Museum of Kansas*, **3**, 62.

Crump, M.L. (1974) Reproductive strategies in a tropical anuran community. *Miscellaneous Publications. University of Kansas*, **61**, 1-68.

Cusi, J.C., Barboza, A.C., Vredenburg, V. & von May, R. (2015) A new locality, range extension and record of *Batrachochytrium dendrobatidis* in the endangered terrestrial breeding frog *Pristimantis katoptroides* Flores, 1988 (Anura: Craugastoridae) in Peru. *Check List*, **11**, 1608.

De la Riva, I.J. (1993) *Ecología de una comunidad neotropical de anfibios durante la estación lluviosa*. Universidad Complutense, Madrid.

Doan, T.M. & Arizábal, W. (2002) Microgeographic variation in species composition of the herpetofaunal communities of Tambopata Region, Peru. *Biotropica*, **34**, 101-117.

Donnelly, M.A., Chen, M.H. & Watkins, G.G. (2005) Sampling amphibians and reptiles in the Iwokrama forest ecosystem. *Proceedings of the Academy of Natural Sciences of Philadelphia*, **154**, 55-69.

Duellman, W.E. (2004) Frogs of the genus *Colostethus* (Anura; Dendrobatidae) in the Andes of northern Peru. *Scientific Papers. Natural History Museum. The University of Kansas*, **35**, 1–49.

Duellman, W.E. (2005) *Cusco Amazónico: The lives of amphibians and reptiles in an Amazonian rainforest*. Cornell University Press., New York.

Duellman, W.E. & Toft, C.A. (1979) Anurans from Serrania de Sira, Amazonian Peru: taxonomy and biogeography. *Herpetologica*, 60-70.

Duellman, W.E. & Trueb, L. (1986) *Biology of Amphibians*. McGraw Hill, New York.

Duellman, W.E. & Lynch, J.D. (1988) Anuran amphibians from the Cordillera de Cutucú, Ecuador. *Proceedings of the Academy of Natural Sciences of Philadelphia*, **140**, 125-142.

Duellman, W.E. & Wiens, J.J. (1993) Hylid frogs of the genus *Scinax* Wagler, 1830, in Amazonian Ecuador and Peru. *Occasional Papers of the Museum of Natural History. University of Kansas*, **153**, 1-57.

Duellman, W.E. & Mendelson, J.R. (1995) Amphibians and reptiles form Northern Departamento Loreto, Perú: taxonomy and biogeography. *University of Kansas Science Bulletin*, **55**, 329-376.

Duellman, W.E. & Thomas, R. (1996) Anuran amphibians from a seasonaly dry forest in southeastern Peru and comparisons of the anurans among sites in the Upper Amazon Basin. *Occasional Papers of the Natural History Museum of Kansas*, **180**, 1-34.

Duellman, W.E. & Lehr, E. (2009) *Terrestrial-breeding frogs (Strabomantidae) in Peru*. Natur und Tier, Verlag GmbH, Science.

Duellman, W.E. & Trueb, L. (2015) *Marsupial frogs:* Gastrotheca *and allied genera*. Johns Hopkins University Press, Baltimore, Maryland, USA.

Dunn, E.R. (1942) The American caecilians. *Bulletin of The Museum of Comparative Zoology*, **91**, 439-540.

Faivovich, J., Haddad, C.F.B., Garcia, P.C.A., Frost, D.R., Campbell, J.A. & Wheeler, W.C. (2005) Systematic review of the frog family Hylidae, with special reference to Hylinae: a phylogenetic analysis and taxonomic revision. *Bulletin of the American Museum of Natural History*, **294**, 1–240.

Fouquet, A., Jean-Pierre, V., Kadosoe, V., Ouboter, P. & Jairam, R. (2015) Checklist of the amphibians of the Sipaliwini area, Suriname. *Herpetology Notes*, **8**, 63-68.

França, F.G.R. & Venâncio, N.M. (2010) Reptiles and amphibians of a poorly known region in southwest Amazonia. *Revista Biotemas*, **23**, 71-84.

Frenkel, C., H., Y.-M.M., Guayasamín, J.M., Varela-Jaramillo, A. & Ron, S.R. (2013) *Pristimantis trachyblepharis*. Available at: http://zoologia.puce.edu.ec/vertebrados/anfibios (accessed 15 May 2016).

Frost, D.R. (2017) *Amphibian species of the World: an online reference. Version 6.0.* Available at: <http://research.amnh.org/herpetology/amphibia/index.php/> (accessed 5 January 2017)

Funk, W.C., Fletcher-Lazo, G., Nogales-Sornosa, F. & Almeida-Reinoso, D. (2004) First Description of a clutch and nest site for the genus *Caecilia* (Gymnophiona: Caeciliidae). *Herpetological Review*, **35**, 128-130.

Guayasamin, J.M. & Funk, W.C. (2009) The amphibian community at Yanayacu Biological Station, Ecuador, with a comparison of vertical microhabitat use among *Pristimantis* species and the description of a new species of the *Pristimantis myersi* group. *Zootaxa*, **2220**, 41-66.

Guayasamin, J.M., Tapia, E., Aldás, S. & Deichmann, J. (2011) Anfibios y reptiles de los tepuyes de la Cuenca Alta del Río Nangaritza, Cordillera del Cóndor. *Evaluación Ecológica Rápida de la biodiversidad de los tepuyes de la Cuenca Alta del Río Nangaritza, Cordillera del Cóndor, Ecuador* (ed. by J.M. Guayasamin and E. Bonaccorso), pp. 56-62. Conservation International, Quito, Ecuador.

Guerrero, M., Venegas, P.J., Gagliardi, G., Suarez, A., Toyama, R., Contreras, V.H. & Ruiz, J. (2011) Anfibios y reptiles. Centro de Investigaciones Jenaro Herrera – Loreto, Peru. *Field Museum Rapid Color Guides*, **286**, 1-10.

Haddad, C.F.B. & Prado, C.P.A. (2005) Reproductive modes in frogs and their unexpected diversity in the Atlantic forest of Brazil. *BioScience*, **55**, 207-217.

Harvey, M.B., Almendáriz, A., Brito, M. & Batallas, D. (2013) A new species of *Noblella* (Anura: Craugastoridae) from the Amazonian slopes of the Ecuadorian Andes with comments on *Noblella lochites* (Lynch). *Zootaxa*, **3635**, 1-14.

Heyer, W.R., García-López, J.M. & Cardoso, A.J. (1996) Advertisement call variation in the *Leptodactylus mystaceus* species complex (Amphibia: Leptodactylidae) with a description of a new sibling species. *Amphibia-Reptilia*, **17**, 7-31.

Hödl, W. (1990) Reproductive diversity in Amazonian lowland frogs. *Fortschritte der Zoologie*, **38**, 41-60.

Jimenez-Robles, O., De la Riva, I. & Guayasamin, J.M. (2015) *Rulyrana flavopunctata* (Yellow-spotted Cochran Frog). Reproduction. *Herpetological Review*, **46**, 238-239.

Jungfer, K.-H. (2011) A new tree frog of the genus *Osteocephalus* from high altitudes in the Cordillera del Cóndor, Ecuador (Amphibia: Anura: Hylidae). *The Herpetological Journal*, **21**, 247-253.

Jungfer, K.H., Faivovich, J., Padial, J.M., Castroviejo‐Fisher, S., Lyra, M.M., Berneck, B., Iglesias, P.P., Kok, P.J., MacCulloch, R.D. & Rodrigues, M.T. (2013) Systematics of spiny‐backed treefrogs (Hylidae: *Osteocephalus*): an Amazonian puzzle. *Zoologica Scripta*, **42**, 351-380.

Köhler, J. (2000) Amphibian diversity in Bolivia: a study with special reference to montane forest regions. *Bonner Zoological Monographs*, **48**, 1–243.

Köhler, J. & Lötters, S. (2001) Description of a small tree frog, genus *Hyla* (Anura: Hylidae), from humid Andean slopes of Bolivia. *Salamandra*, **37**, 175-184.

Krügel, P. & Richter, S. (1995) *Syncope antenori*: a bromeliad breeding frog with free-swimming, nonfeeding tadpoles (Anura, Microhylidae). *Copeia*, 955-963.

Lehr, E. & Duellman, W.E. (2009) *Terrestrial-breeding frogs (Strabomantidae) in Peru*. Natur und Tier Verlag, Múnster, Germany.

Lima, A.P., Magnusson, W.E., Menin, M., Erdtmann, L.K., Rodrigues, D.J., Keller, C. & Hödl, W. (2006) *Guia de sapos da Reserva Adolpho Ducke, Amazônia Central. Manaos*. Áttema Design Editorial, Manaos.

Lujan, L., Venegas, P.J. & Echevarría, L.Y. (2014) *Cochranella nola* Harvey, 1996 (Amphibia, Anura, Centrolenidae): First country record from Peru. *Herpetology Notes*, **7**, 607-608.

Lynch, J.D. (2005) Discovery of the richest frog fauna in the world—an exploration of the forests to the north of Leticia. *Revista de la Academia Colombiana de Ciencias*, **29**, 581-588.

Lynch, J.D. & Duellman, W.E. (1980) The *Eleutherodactylus* of the Amazonian slopes of the Ecuadorian Andes (Anura: Leptodactylidae). *Miscellaneous Publications. University of Kansas*, **69**, 1-86.

Middendorf, G. & Reynolds, R. (2000) Herpetofauna of the Beni Biological Station Biosphere Reserve, Amazonian Bolivia: additional information, and current knowledge in context. *Biodiversity, conservation and management in the region of Beni Biological Station Biosphere Reserve, Bolivia* (ed. by O. Herrera Macbryde, F. Dallmeier, B. Macbryde, J.A. Comiskey and C. Miranda), pp. 129-150. Smithsonian Institution/MAB Biodiversity Program, Washington, D.C.

Mueses-Cisneros, J.J. (2005) The amphibian fauna of the Valle de Sibundoy, Putumayo-Colombia. *Caldasia*, **27**, 229-242.

Mueses-Cisneros, J.J., Cisneros-Heredia, D.F. & McDiarmid, R.W. (2010) A New Amazonian species of *Rhaebo* (Anura: Bufonidae) with comments on *Rhaebo glaberrimus* (Günther, 1869) and *Rhaebo guttatus* (Schneider, 1799). *Zootaxa*, **3447**, 22-40.

Neckel-Oliveira, S. & Gordo, M. (2004) Anfíbios, Lagartos e serpentes do Parque Nacional do Jaú. *Janelas para a biodiversidade no Parque Nacional do Jaú: uma estratégia para o estudo da biodiversidade na Amazônia* (ed. by S.H. Borges, S. Iwanaga, C.C. Durigan and M.R. Pinheiro), pp. 161-176. Fundaçao Vitória Amazônica, Manaus, Brasil.

Ortega-Andrade, M. (2010) *Diversidad de la herpetofauna en la Centro Amazonía de Ecuador*. Universidad Nacional Autónoma de México, Xalapa, Veracruz.

Ouboter, P.E., Jairam, R. & Kasanpawiro, C. (2011) A rapid assessment of the amphibians and reptiles of the Kwamalasamutu region (Kutari/lower Sipaliwini Rivers), Suriname. *A Rapid Biological Assessment of the Kwamalasamutu region, Southwestern Suriname* (ed. by B.J. O'shea, L.E. Alonso and T.H. Larsen), pp. 124-130. Conservation International, Arlington, VA.

Padial, J.M., Chaparro, J.C., Castroviejo-Fisher, S., Guayasamin, J.M., Lehr, E., Delgado, A.J., Vaira, M., Teixeira Jr, M., Aguayo, R. & Riva, I.d.l. (2012) A revision of species diversity in the Neotropical genus *Oreobates* (Anura: Strabomantidae), with the description of three new species from the Amazonian slopes of the Andes. *American Museum Novitates*, 1-55.

Paez-Vacas, M.I., Coloma, L.A. & Santos, J.C. (2010) Systematics of the *Hyloxalus bocagei* complex (Anura: Dendrobatidae), description of two new cryptic species, and recognition of *H. maculosus*. *Zootaxa*, **2711**, 1-75.

Pereyra, M.O., Candioti, M.F.V., Faivovich, J. & Baldo, D. (2015) Egg clutch structure of *Rhinella rumbolli* (Anura: Bufonidae), a toad from the Yungas of Argentina, with a review of the reproductive diversity in *Rhinella*. *Salamandra*, **51**, 161-170.

Pérez, P., Bodmer, R. & Puertas, P. (2006) Anuros y saurios del Interfluvio Yavarí–Tahuayo y su comparación con las Áreas Naturales Protegidas en la Región Loreto, Perú. *Memoria VI Congreso Internacional sobre Manejo de Fauna Silvestre en la Amazonía y Latinoamérica*, 1-15.

Pinheiro, L.C., Bitar, Y., Galatti, U., Neckel-Oliveira, S. & Santos-Costa, M. (2012) Amphibians from southeastern state of Pará: Carajás Region, northern Brazil. *Check List*, **8**, 693-702.

Prudente, A.L.C., Sturaro, M.J., Travassos, A.E.M., Maschio, G.F. & Costa, M.C.S. (2013) Anurans of the Urucu Petrol Basin, municipality of Coari, State of Amazonas, northern Brazil. *Check List*, **9**, 601-606.

Ramalho, W.P., Andrade, M.S., Matos, L.R.A.d. & Vieira, L.J.S. (2016) Amphibians of varzea environments and floating meadows of the oxbow lakes of the Middle Purus River, Amazonas, Brazil. *Biota Neotropica*, **16**

Ramírez, S., Meza-Ramos, P., Yánez-Muñoz, M. & Reyes, J. (2009) Asociaciones interespecíficas de anuros en cuatro gradientes altitudinales de la Reserva Biológica Tapichalaca, Zamora-Chinchipe, Ecuador. *Serie Zoológica*, **4**, 35-49.

Reichle, S. (2007) *Distribution, diversity and conservation status of Bolivian Amphibians*. Universitäts und Landesbibliothek Bonn,

Reyes-Puig, J.P., Reyes-Puig, C., Pérez, M.B. & Yánez-Muñoz, M.H. (2015) Dos nuevas especies de ranas *Pristimantis* (Craugastoridae) de la cordillera de los Sacha Llanganatis, vertiente oriental de los Andes de Ecuador. *Avances en Ciencias e Ingenierías*, **7**, B61-B74.

Reyes-Puig, J.P., Reyes-Puig, C., Rámirez-Jaramillo, S.R., Pérez, M.B. & Yánez-Muñoz, M.H. (2014) Tres nuevas especies de ranas terrestres *Pristimantis* (Anura: Craugastoridae) de la cuenca alta del Río Pastaza, Ecuador. *Avances en Ciencias e Ingenierías*, **6**, B51-B62.

Reyes-Puig, M., Reyes-Puig, J. & Yánez-Muñoz, M. (2013) Ranas terrestres del género *Pristimantis* (Anura: Craugastoridae) de la Reserva Ecológica Río Zúñag, Tungurahua, Ecuador: Lista anotada y descripción de una especie nueva. *Avances en Ciencias e Ingenierías*, **5**, B5-B13.

Reynolds, R., Fritts, T., Gotte, S., Icochea, J. & Tello, G. (1997) Amphibians and reptiles. *Biodiversity Assessment and Long-term Monitoring of the Lower Urubamba Region, Peru: San Martin-3 and Cashiriari-2 Well Sites* (ed. by F. Dallmeier and A. Alonso), pp. 129-150. Smithsonian Institution/MAB Biodiversity Program, Washington, D.C.

Rödder, D. & Schlüter, A. (2009) *Pristimantis minutulus* Duellman & Hedges, 2007 (Anura: Strabomantidae): geographic range extension and colour polymorphism. *Salamandra*, **45**, 53-56.

Rodriguez, L.O. & Knell, G. (2003) Anfibios y reptiles. *Perú: Yavarí* (ed. by N. Pitman, C. Vriesendorp and D.K. Moskovits), pp. 63-67. The Field Museum, Chicago.

Rodríguez, L.O. ( 2001) The herpetofauna of the Northern Cordillera de Vilcabamba, Peru. *Biological and Social Assessments of the Cordillera de Vilcabamba, Peru* (ed. by L.E. Alonso, A. Alonso, T.S. Schulenberg and F. Dallmeier), pp. 127-130. Conservation Internacional, Washington.

Rodríguez, L.O. & Cadle, J.E. (1990) A preliminary overview of the herpetofauna of Cocha Cashu, Manu National Park, Peru. *Four Neotropical Rainforests* (ed. by A.H. Gentry), pp. 410-425. Yale University Press, New Haven, Connecticut, USA.

Rodríguez, L.O. & Duellman, W.E. (1994) Guide to the frogs of the Iquitos Region, Amazonian Peru. *University of Kansas Natural History Museum Special Publications*, **22**, 1-80.

Rodríguez, L.O. & Catenazzi, A. (2004) Anfibios y reptiles. *Perú: Megantoni* (ed. by C. Vriesendorp, L. Rivera Chávez, D.K. Moskovits and J. Shopland), pp. 92-98. The Field Museum, Chicago.

Rodríguez, L.O., Pérez Z., J. & Shaffer, H.B. (2001) Anfibios y reptiles. *Perú: Biabo, Cordillera Azul* (ed. by W.S. Alverson, L.O. Rodríguez and D.K. Moskovits), pp. 69-75. The Field Museum, Chicago.

Ron, S.R. & Pramuk, J.B. (1999) A new species of *Osteocephalus* (Anura: Hylidae) from Amazonian Ecuador and Peru. *Herpetologica*, **55**, 433-446.

Ron, S.R., Guayasamin, J.M., Yanez-Muñoz, M.H., Merino-Viteri, A., Ortiz, D.A. & Nicolalde, D.A. (2015) *AmphibiaWebEcuador. Version 2015.0. Museo de Zoología, Pontificia Universidad Católica del Ecuador.* Available at: http://zoologia.puce.edu.ec/Vertebrados/anfibios. (accessed 6 December 2015).

Schlüter, A., Icochea, J. & Perez, J. (2004) Amphibians and reptiles of the lower Río Llullapichis, Amazonian Peru: updated species list with ecological and biogeographical notes. *Salamandra*, **40**, 141-160.

Schlüter, A., Löttker, P. & Mebert, K. (2009) Use of an active nest of the leaf cutter ant *Atta cephalotes* (Hymenoptera: Formicidae) as a breeding site of *Lithodytes lineatus* (Anura: Leptodactylidae). *Herpetology Notes*, **2**, 101-105.

Souza, M.B. ( 2009) *Anfíbios – Reserva Extrativista do Alto Juruá e Parque Nacional da Serra do Divisor, Acre*. Unicamp, Campinas, Brasil.

Suárez-Mayorga, A.M. (1999) Lista preliminar de la fauna Amphibia presente en el transecto La Montañita-Alto de Gabinete, Caquetá, Colombia. *Revista de la Academia Colombiana de Ciencias*, **23**, 395-405.

Trueb, L. (1971) Phylogenetic relationships of certain neotropical toads with the description of a new genus (Anura: Bufonidae). *Contributions in Science*, **216**, 1–40.

Upton, K.A. (2015) *Amphibian diversity in Amazonian flooded forests of Peru*. Institute of Conservation and Ecology, School of Anthropology and Conservation, University of Kent,

Venegas, P.J., Gagliardi-Urrutia, G. & Odicio, M. (2014) Amphibians and reptiles. *Perú: Cordillera Escalera-Loreto. Rapid Biological and Social Inventories Report 26.* (ed. by N. Pitman, C. Vriesendorp, D. Alvira, J.A. Markel, M. Johnston, E.R. Inzunza, A.L. Pizango, G.S. Valenzuela, P. Álvarez-Loayza, T.W. J. Homan, Á.D. Campo, D.F. Stotz, S. Heilpern and C. The Field Museum), pp. 319–329. The Field Museum, Chicago.

Vigle, G.O. (2008) The amphibians and reptiles of the Estación Biológica Jatún Sacha in the lowland rainforest of Amazonian Ecuador: a 20-year record. *Breviora*, **514**, 1-30.

von May, R., Siu-Ting, K., Jacobs, J.M., Medina Müller, M., Gagliardi, G., Rodríguez, L.O. & Donnelly, M.A. (2009) Species diversity and conservation status of amphibians in Madre de Dios, Southern Peru. *Herpetological Conservation and Biology*, **4**, 14-29.

Waldez, F., Menin, M. & Vogt, R.C. (2013) Diversidade de anfíbios e répteis Squamata na região do baixo rio Purus, Amazônia Central, Brasil/Diversity of amphibians and Squamata reptilians from lower Purus River Basin, Central Amazonia, Brazil. *Biota Neotropica*, **13**, 300.

Withworth, A. & Villacampa-Ortega, J. (2015) Amphibians of the Manu Learning Centre. Rio Alto Madre de Dios – 450-750m asl, Madre de Dios, SE Perú. Versión 1.1. *Field Museum Rapid Color Guides*, **645**, 1–8.

Yánez-Muñoz, M.H. & Reyes-Puig, J.P. (2008) Evaluación de la herpetofauna de las reservas biológicas de la Fundación Ecominga. *Informe técnico División de Herpetología Museo Ecuatoriano de Ciencias Naturales*, **25**, 1–62.

Zimmerman, B.L. & Rodrigues, M.T. (1990) The frogs, snakes, and lizards of the INPA-WWF reserves near Manaus in the central Amazon. *Four neotropical rain forests* (ed. by A.H. Gentry), pp. 426-454. Yale University Press, New Haven.

Zimmerman, B.L. & Simberloff, D. (1996) An historical interpretation of habitat use by frogs in a Central Amazonian forest. *Journal of Biogeography*, **23**, 27-46.
